# Unfolding Plant Defence: Endoplasmic Reticulum Stress Signalling at the Plant‐Pathogen Interface

**DOI:** 10.1111/pbi.70662

**Published:** 2026-04-11

**Authors:** Zhe Meng, Shuqin Zheng, Federica Brandizzi, Yiran Liu, Chunlei Li, Congcong Ai, Yujiao Wang, Jiadong Qi, Xiuguo Zhang

**Affiliations:** ^1^ Shandong Provincial Key Laboratory of Plant Stress Biology and Genetic Improvement, College of Life Sciences Shandong Normal University Jinan China; ^2^ Plant Research Laboratory Michigan State University East Lansing Michigan USA; ^3^ Shandong Provincial Key Laboratory for Biology of Vegetable Diseases and Insect Pests, College of Plant Protection Shandong Agricultural University Tai'an China

**Keywords:** effector‐triggered immunity, endoplasmic reticulum stress, ER‐quality control, pathogen effector, pattern‐triggered immunity, plant immunity, unfolded protein response

## Abstract

The endoplasmic reticulum (ER) stress response, a conserved proteostasis network, has emerged as a central hub that reprograms plant immunity during pathogen attack. This review synthesises how plants harness ER‐stress signalling to mount multilayered defences and how pathogens have evolved counterstrategies to subvert these pathways. We delineate the molecular integration of the unfolded protein response (UPR) with canonical immune layers including pattern‐triggered immunity (PTI), effector‐triggered immunity (ETI) and systemic defences, highlighting salicylic acid (SA) and jasmonic acid (JA) as rheostats that fine‐tune ER stress‐immune crosstalk. Functionally, the UPR bolsters immunity by coordinating protein folding and secretion, reprogramming transcription and translation, activating ER‐dependent programmed cell death (ER‐PCD), and orchestrating ER‐associated degradation (ERAD) and selective autophagy. Pathogens such as bacteria, oomycetes and viruses in turn deploy virulence factors that target UPR sensors and transcription factors, thereby attenuating ER‐driven immunity. We propose a conceptual framework in which the outcome of UPR activation—resistance versus susceptibility—is determined by pathogen lifestyle, ER stress dynamics, subcellular compartmentalisation and pathogen effector intervention. We also consider biotechnological contexts in which strong transgene expression can itself provoke the UPR, and outline diagnostic experimental strategies to distinguish UPR‐mediated effects from intended transgene functions. By integrating molecular mechanisms with pathogen counterstrategies, this review underscores the dynamic interplay between ER stress and immune signalling in plants and highlights opportunities to enhance crop resilience under global climate challenges.

## 1 Introduction

As sessile organisms, plants constantly face biotic stressors, including a wide range of pathogens such as bacteria, fungi, oomycetes and viruses. To cope with these, plants have developed a multilayered immune system that integrates classical defence signalling with organelle‐specific stress responses. Among these organelles, the endoplasmic reticulum (ER) acts as a pivotal hub. ER‐dependent stress‐signalling networks couple proteostasis surveillance to the activation of pathogen‐responsive pathways. Beyond its canonical roles in protein synthesis, folding, and secretion, the ER orchestrates multiple signal‐transduction cascades that fine‐tune both basal and inducible plant immunity (Breeze et al. 2020).

**FIGURE 1:**
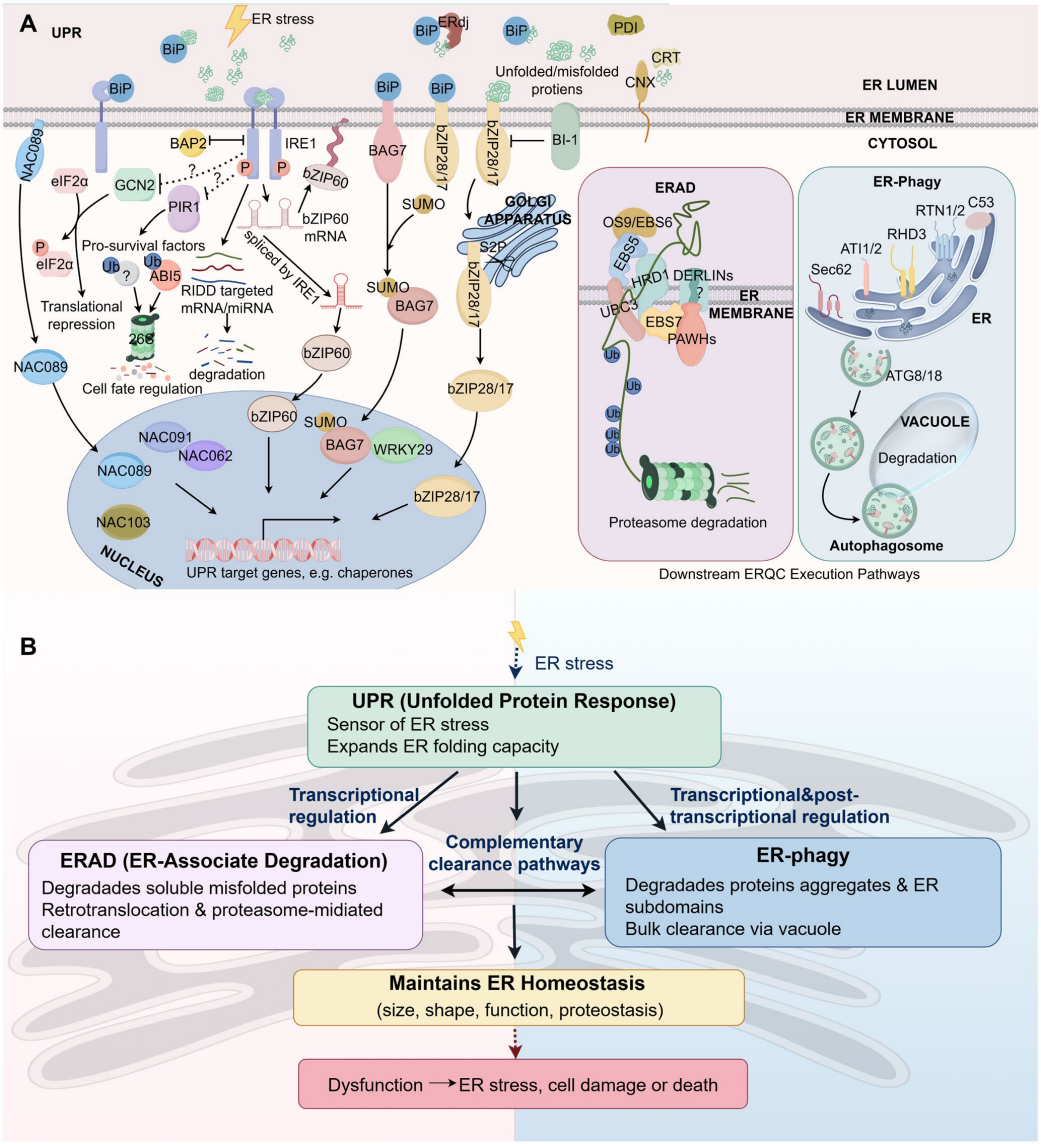
The plant ER quality control (ERQC) network. (A) Core UPR signalling branches and their interconnection with ERAD and ER‐phagy. Newly synthesised proteins are folded by ER‐resident chaperones, including binding immunoglobulin protein (BiP), calnexin (CNX), calreticulin (CRT), protein disulfide isomerase (PDI) and ERdj co‐chaperones. Accumulation of unfolded/misfolded proteins causes BiP to dissociate from the membrane sensors IRE1 and bZIP28/17, activating two parallel signalling branches. Activated‐IRE1 catalyses unconventional splicing of *bZIP60* mRNA and executes regulated IRE1‐dependent decay (RIDD) of mRNAs and miRNAs. IRE1 also restrains GCN2‐eIF2α hyperactivation to preserve translational homeostasis and inhibits the pro‐death E3 ligase PIR1, thereby stabilising the survival factor ABI5. Concurrently, bZIP28/17 translocates to the Golgi for proteolytic activation, after which its cytosolic domain enters the nucleus. Nuclear bZIP60 and bZIP28 cooperate with NAC transcription factors (NAC089, NAC103, NAC062, NAC091) to drive UPR target gene expression, including ER chaperones. Under ER stress, BAG7 dissociates from BiP and bZIP28, undergoes modification, and relocates to the nucleus, where it cooperates with WRKY29 to activate chaperone transcription. BAP2 monitors IRE1‐mediated signalling through a negative feedback loop, while BI‐1 restrains the pro‐adaptive activity of bZIP28, modulating the balance between survival and death. Under sustained stress, NAC089 promotes programmed cell death (PCD). ERAD (via HRD1, EBS5/6/7, UBC32, and PAWHs) retrotranslocates misfolded proteins for proteasomal degradation, whereas ER‐phagy (mediated by ATI1/2, Sec62, C53, RTN1/2 and RHD3) clears protein aggregates and ER subdomains through vacuolar delivery. (B) Functional hierarchy and crosstalk among UPR, ERAD and ER‐phagy during ER stress. ER stress activates the UPR, which expands folding capacity through transcriptional and post‐transcriptional reprogramming. ERAD and ER‐phagy serve as complementary clearance modules. Dysfunction of any component leads to persistent ER stress, cellular damage, and death.

Because the ER is intrinsically sensitive to pathogen‐induced perturbations, even modest disturbances can destabilise its folding environment. When adverse conditions increase the demand for secretory defence products such as pathogenesis‐related (PR) proteins and antimicrobial peptides beyond the ER's folding capacity, unfolded and misfolded polypeptides accumulate, triggering ER stress. If unmitigated, ER stress can be lethal. To re‐establish ER homeostasis, plants activate an integrated ER quality‐control (ERQC) network (Figure 1A). The unfolded protein response (UPR) serves as the central regulatory hub of this network, coordinating two complementary downstream execution pathways: ER‐associated degradation (ERAD) for targeted proteasomal clearance of misfolded proteins, and ER‐phagy for selective autophagic turnover of damaged ER subdomains (Figure 1B) (Molinari 2021; Duan et al. 2023; Bao and Bassham 2020). Together, these conserved response modules either retain aberrant proteins for refolding or route them to degradation. If homeostasis cannot be restored—owing to sustained or overwhelming stress—the UPR circuitry shifts toward a programmed cell‐death (PCD) pathway that sacrifices compromised cells to confine pathogen proliferation (Simoni et al. 2022).

Mounting evidence indicates that the UPR is tightly interwoven with plant immune circuitry, underscoring a dynamic cross‐talk between ER stress signalling and pathogen‐defence modules. During microbial assault, UPR activation can shift the balance toward either susceptibility or resilience, depending on cellular context and the nature of the stressor (Inaba et al. 2023). On the protective side, ER‐derived signals reinforce immunity by coordinating the production and secretion of defence proteins, priming PCD to confine invading microbes, and fine‐tuning phytohormone networks such as salicylic acid (SA) and jasmonic acid (JA) (Breeze et al. 2020; Carvalho et al. 2014; Wang et al. 2005). In contrast, pathogens hijack the ER stress response to remodel host physiology, attenuating immune outputs and promoting infection (Jing et al. 2016; Jing and Wang 2020). Collectively, these observations raise a central question: how do plants calibrate ER‐stress signalling to maximise defensive gain while thwarting pathogenic takeover?

This review delineates the multifaceted roles of ER‐stress signalling in plant defences against biotic stress, emphasising its crosstalk with both innate and acquired immune circuits. We begin by detailing the molecular architecture of the plant ERQC network, with a focus on the UPR as its central regulatory hub. We then chart how this UPR‐centered network interfaces with PAMP‐triggered immunity (PTI), effector‐triggered immunity (ETI), and the ensuing defence cascades. We then interrogate how ER‐resident sensors and effectors modulate immune‐protein trafficking, transcriptional reprogramming, PCD, ERAD and ER‐phagy during infection. In addition, we examine the dual role of ER stress as both a fortification of host defence and a liability that can be commandeered by pathogens, and we synthesise these observations into a conceptual framework that explains context‐dependent UPR outcomes. Furthermore, we explore the implications of ER‐stress signalling for crop biotechnology, outlining key challenges and future directions.

## 2 The ER Quality Control Network in Plants

### 2.1 The Conserved UPR Network in Plants: A Brief Overview

In plants, the core UPR machinery has been extensively characterised in the model organism *Arabidopsis thaliana* , where it primarily comprises two evolutionarily conserved signalling branches mediated by IRE1‐bZIP60 and bZIP28/bZIP17, respectively (Figure 1A) (Liu and Howell 2010; Deng et al. 2011; Song et al. 2015). Core components of the UPR are evolutionarily conserved and functionally essential across diverse vascular plant species, highlighting the fundamental role of the UPR in plant biology (Howell 2021). The ER‐resident chaperone binding immunoglobulin protein (BiP) acts as a master regulator for both branches, sensing luminal stress and activating the transmembrane sensors IRE1 and bZIP28/bZIP17 to initiate signalling. Activated IRE1, an ER‐localised kinase/endoribonuclease, executes two principal functions: (i) it catalyses the unconventional splicing of *bZIP60* mRNA, generating an active transcription factor that induces UPR target genes; and (ii) it degrades selected mRNAs encoding secretory proteins through regulated IRE1‐dependent decay (RIDD), thereby reducing the ER client load to promote cell survive or, alternatively, degrading protective transcripts to initiate programmed cell death (PCD) (Bao et al. 2018; Mishiba et al. 2013). Recent studies have substantially expanded the understanding of RIDD substrate specificity, identifying key developmental regulators such as *RAPTOR1b* and specific microRNAs (e.g., miR172) as novel targets (Reagan et al. 2025; Li, Qin, et al. 2025). This broad yet selective targeting enables IRE1‐mediated RNA decay to extend beyond acute stress mitigation to developmental homeostasis. Beyond its RNA‐related functions, IRE1 has emerged as a guardian of translational homeostasis, as its loss causes severe global translation suppression under ER stress, in part through hyperactivation of the GCN2‐eIF2α axis (Yoo et al. 2024). IRE1 also dictates cell fate by interfacing with the ubiquitin‐proteasome system: it negatively regulates the pro‐death E3 ligase PIR1 to stabilise the survival factor ABI5 (Ko et al. 2023; Varshney et al. 2024), while controlling BAP2 levels through negative feedback to prevent UPR overactivation (Pastor‐Cantizano et al. 2024). Thus, IRE1 functions as a multidimensional signalling hub integrating transcriptional, post‐transcriptional, translational and proteostatic outputs. The second UPR branch is initiated by the ER‐membrane‐anchored transcription factors bZIP28 and bZIP17, which translocate to the Golgi for proteolytic activation. The liberated cytosolic domain enters the nucleus to activate the expression of stress‐responsive genes, such as chaperones and foldases (Liu et al. 2007a; Srivastava et al. 2013, 2012, 2014; Iwata et al. 2017).

The transcriptional output of these branches is amplified and diversified by NAC transcription factors (e.g., NAC089, NAC103, NAC062, NAC091), which are differentially induced and regulate subsets of genes involved in adaptation or PCD (Yang et al. 2023; Yang, Lu, et al. 2014; Sun, Yang, Song, et al. 2013; Yang, Wang, et al. 2014). Cell fate decisions under sustained ER stress are further fine‐tuned by modulators such as Bax Inhibitor‐1 (BI‐1) and Bcl‐2‐associated athanogene 7 (BAG7), which interface with the core UPR machinery to influence survival versus death outcomes (Hetz et al. 2006; Watanabe and Lam 2008b; Williams et al. 2010; Li et al. 2017). Ultimately, the execution of the UPR relies on a suite of ER chaperones and foldases that mediate protein folding, assembly, and quality control. Key among these are the Hsp70 family member BiP (Srivastava et al. 2013), the lectin‐like chaperones calnexin (CNX) and calreticulin (CRT) (Hammond and Helenius 1994; Ruddock and Molinari 2006), protein disulfide isomerases (PDIs) (Braakman and Bulleid 2011), and DnaJ‐family co‐chaperones (ERdj) (Jin et al. 2009, 2008).

The intricate interplay among these core components, co‐factors, and chaperones constitutes a robust network for ER homeostasis control (Duwi Fanata et al. 2013; Howell 2013, 2021).

### 2.2 The ERQC Triad: UPR as the Master Regulator of ERAD and ER‐Phagy

Beyond its direct role in enhancing ER folding capacity, the UPR functions as the central regulatory hub of a broader ERQC network (Figure 1). Mechanistically, the UPR exerts multi‐layered transcriptional and post‐transcriptional control over both ERAD and ER‐phagy pathways. UPR‐activated transcription factors—including bZIP60, bZIP28, bZIP17 and NACs—directly upregulate distinct sets of ERQC components. They induce the expression of key ERAD genes (e.g., the E3 ligases *HRD1A/B*, the adaptors *OS9/EBS6* and *HRD3A/EBS5*, and the ubiquitin‐conjugating enzymes *UBC3*) (Deng et al. 2011; Liu et al. 2007b, 2007a; Kamauchi et al. 2005; Martínez and Chrispeels 2003), while simultaneously activating ER‐phagy receptors (e.g., *Sec62*, *ATI1/ATI2*, *RHD3*, *C53*) and autophagy‐related machinery (Figure 1A) (Hu et al. 2020; Honig et al. 2012; Wu et al. 2021; Sun et al. 2022; Stephani et al. 2020). Moreover, the UPR sensor IRE1 also regulates ER‐phagy post‐transcriptionally—via RIDD‐mediated decay of mRNAs encoding autophagy repressors (e.g., BGLU21, ROSY1/ML and PR‐14), thereby lifting the brake on autophagy (Bao et al. 2018). Recent evidence further extends IRE1's regulatory reach to the transcriptional control of ERAD: under ER stress, IRE1 is essential for the timely and robust induction of core ERAD component genes, including *HRD1B*, *EBS5*/*HRD3* and *EBS6*/*OS9* (Yoo et al. 2025). Loss of *IRE1* function delays ERAD gene activation, impairs clearance of misfolded clients such as the brassinosteroid receptor Bri1‐5, and, when combined with high misfolded protein load, triggers a pathological feedback suppression that blunts subsequent UPR and ERAD induction (Yoo et al. 2025). Building upon this framework of UPR‐centred ERQC coordination, we next explore how these interconnected modules are mobilised during plant‐pathogen interactions.

## 3 Integration of ER Stress Signalling With Layered Plant Immunity

**FIGURE 2:**
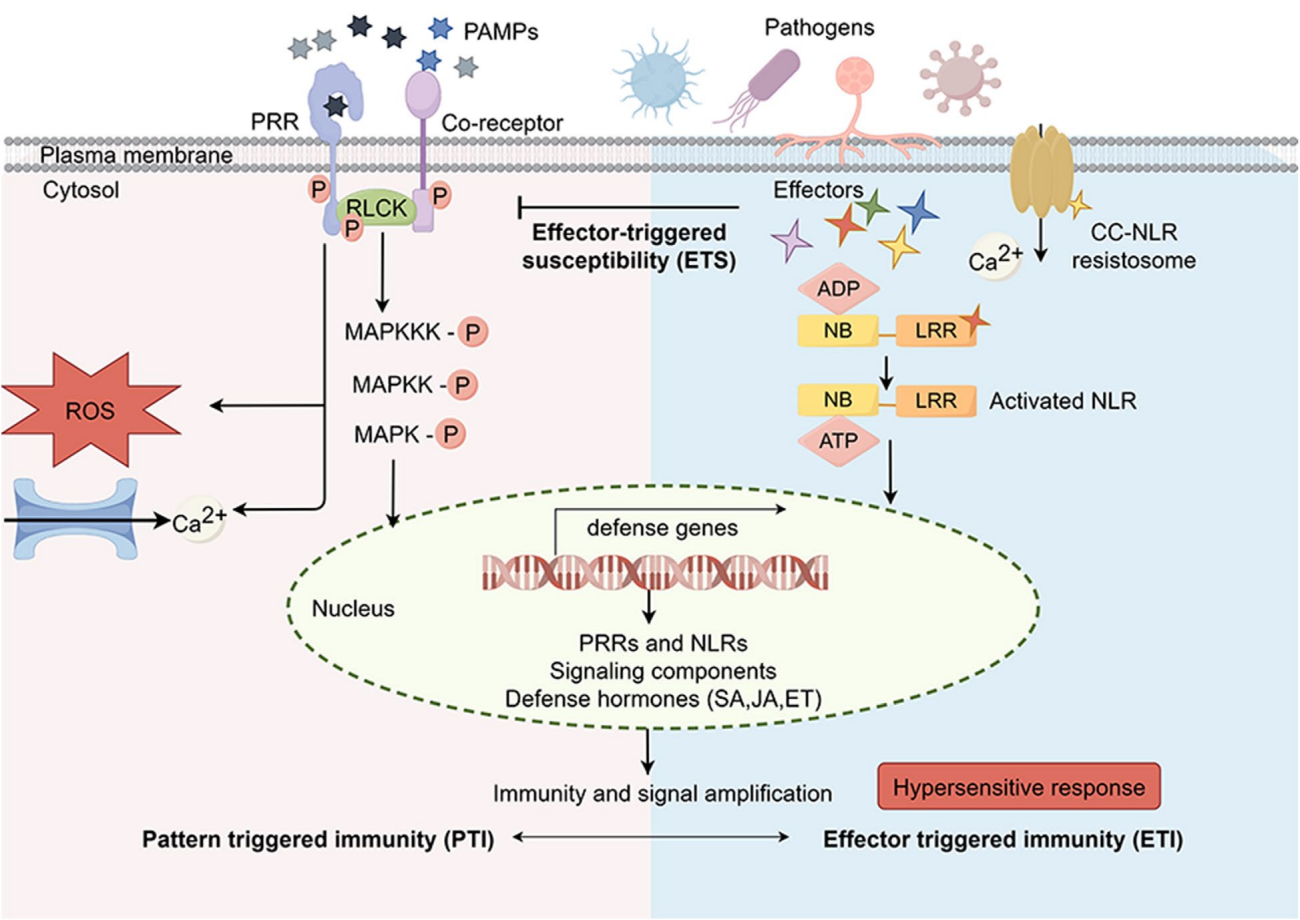
Schematic of the plant innate immune system. The two‐layered architecture of plant immunity is depicted. In the first layer, pathogen‐associated molecular patterns (PAMPs) are recognised by cell‐surface pattern recognition receptors (PRRs) and their co‐receptors, activating receptor‐like cytoplasmic kinases (RLCKs), mitogen‐activated protein kinase (MAPK) cascades, calcium (Ca^2+^) influx, and reactive oxygen species (ROS) bursts to establish pattern‐triggered immunity (PTI). Adapted pathogens deliver effector proteins to suppress PTI, a state termed effector‐triggered susceptibility (ETS). In response, intracellular nucleotide‐binding leucine‐rich repeat (NLR) receptors recognise specific effectors, often via nucleotide exchange (ADP to ATP) and activate effector‐triggered immunity (ETI), frequently culminating in the hypersensitive response (HR). PTI and ETI are interconnected and mutually reinforcing, sharing downstream signalling components and defence outputs. Local immune activation can further induce systemic acquired resistance (SAR) via salicylic acid (SA) and induced systemic resistance (ISR) via jasmonic acid (JA) and ethylene (ET).

To contextualise the interplay with ER stress, it is essential to recall the fundamental architecture of plant immunity. Plants employ a two‐tiered innate immune system (Chisholm et al. 2006; Ngou et al. 2022; Jones and Dangl 2006) (Figure 2). The first layer, pattern‐triggered immunity (PTI), is activated upon recognition of conserved pathogen‐associated molecular patterns (PAMPs) by cell‐surface pattern recognition receptors (PRRs), leading to downstream defence responses. Adapted pathogens secrete effector proteins to suppress PTI, a state known as effector‐triggered susceptibility (ETS). In response, plants have evolved intracellular resistance (R) proteins like nucleotide‐binding leucine‐rich repeat (NLR) receptors that recognise specific effectors, activating a more robust second layer termed effector‐triggered immunity (ETI), often culminating in a hypersensitive response (HR). PTI and ETI are not independent but are interdependent and mutually reinforcing, with their downstream immune responses remarkably overlapping, suggesting a connectivity and convergence between these two layers of defence (Yuan et al. 2023, 2021; Bernoux et al. 2022). Successful local immune activation can induce long‐lasting systemic acquired resistance (SAR), primarily mediated by salicylic acid (SA), or induced systemic resistance (ISR), governed by jasmonic acid (JA) and ethylene (ET) (Balakireva and Zamyatnin 2018; Muthamilarasan and Prasad 2013; Robert‐Seilaniantz et al. 2007; Glazebrook 2005; Durrant and Dong 2004; Choudhary et al. 2007; Heil and Bostock 2002). Mounting evidence reveals that the ER and its associated stress response are not passive compartments but active signalling hubs that engage with PTI, ETI, SAR and ISR to orchestrate effective defence.

### 3.1 Elicitation of the UPR as an Integral Component of Plant Immunity

Both canonical arms of this ER‐stress pathway have been implicated in plant reactions to a remarkably broad spectrum of pathogens—including viruses, bacteria, fungi, and oomycetes.

The IRE1‐bZIP60 branch is indispensable during infection. Transcripts of *IRE1* and *bZIP60* accumulate rapidly after *Pseudomonas* challenge or SA treatment (Moreno et al. 2012; Tateda et al. 2008). Although the two *Arabidopsis* isoforms *IRE1a* and *IRE1b* are co‐induced, they perform only partially overlapping, *bZIP60*‐dependent and ‐independent functions in immunity. The *ire1a ire1b* double mutants, as well as *bzip60* nulls, are hypersusceptible to *P. syringae* pv. *maculicola* ES4326 (Moreno et al. 2012). Whereas *ire1a* plants permit enhanced bacterial multiplication, *ire1b* mutants behave like wild type (Moreno et al. 2012). IRE1a preferentially catalyses bZIP60 mRNA splicing after pathogen attack, whereas IRE1b dominates bZIP60 processing under tunicamycin (Tm)‐induced ER stress (Moreno et al. 2012). Consistently, the IRE1‐bZIP60 module is activated and confers resistance to a range of fungi and viruses, including *Botrytis cinerea* (Blanchard et al. 2025), *Alternaria alternata* (Xu et al. 2019; Blanchard et al. 2025), *Drechslera gigantea* (Samperna et al. 2021), *Plantago asiatica mosaic virus* (PlAMV) (Gayral et al. 2020; Adhikari et al. 2024), *Turnip mosaic virus* (TuMV) (Gayral et al. 2020), *Potato Y virus* (PVY) (Gaguancela et al. 2016) and *Potato X virus* (PVX) (Gaguancela et al. 2016; Ye et al. 2011).

The second UPR branch, governed by membrane‐anchored bZIP28/bZIP17, is likewise required for disease control. Loss of bZIP28 function increases susceptibility to *Tobacco mosaic virus* (TMV) and *Cucumber mosaic virus* (CMV) in *Nicotiana benthamiana* (Shen et al. 2017; Li et al. 2018) and to TMV and *P. syringae* pv. *tomato* (*Pst*) in *Arabidopsis* (Gayral et al. 2020; Arraño‐Salinas et al. 2018). Similarly, bZIP17 appears to exert direct antiviral activity against certain viruses such as PlAMV, as *bzip17* mutants show enhanced susceptibility (Gayral et al. 2020). In each case, pathogen invasion elevates *bZIP28* or *bZIP17* expression (Shen et al. 2017; Li et al. 2018; Gayral et al. 2020). Notably, both *bZIP28* and *bZIP60* are indispensable for plant defence, as highlighted by the susceptibility of single mutants of *Arabidopsis* to *Phytophthora parasitica* (Qiang et al. 2021), and the severely compromised phenotype of the *bzip28 bzip60* double mutant upon *D. gigantea* infection, which includes enlarged lesions, excessive ROS, heightened ion leakage and lipid peroxidation (Samperna et al. 2021).

Additional UPR components also sculpt immune outputs. Viral attack can trigger alternative transcriptional cascades involving BAG7, NAC089, NAC103 and BI‐1 (Gayral et al. 2020; Li et al. 2018; Gaguancela et al. 2016). Genetic evidence demonstrates that BAG7, NAC089 and BI‐1 limit virus accumulation (Gayral et al. 2020; Shen et al. 2017; Li et al. 2018; Gaguancela et al. 2016; Adhikari et al. 2024). *NAC089* is further induced by *Phytophthora* culture filtrate and enhances resistance to *Phytophthora capsici* and *Pst* DC3000 (Ai et al. 2021). Proteomic surveys reveal that molecular chaperones constitute the largest upregulated category during defence, with SGT1, HSP90, HSP70, PDI, ERp57, P5, CRT3, GRP78 and BiP5 among the most prominent (Caplan et al. 2009). Many of these chaperones are synthesised early after pathogen perception, preceding the secretion of defence proteins (Janssens et al. 2014). Disrupting their function compromises immunity and hampers the production of defence‐related proteins (Li et al. 2009; Saijo et al. 2009; Häweker et al. 2010; Nekrasov et al. 2009); for example, *crt3, erdj3b* and *uggt* null mutants exhibit heightened susceptibility to *Pst* DC3000 (Li et al. 2009; Häweker et al. 2010; Nekrasov et al. 2009).

Thus, although distinct UPR‐linked factors participate in pathogen‐specific responses, current evidence strongly supports UPR activation as a universal hallmark of plant immune reprogramming, perhaps by facilitating the maturation and folding of secreted proteins involved in defence.

### 3.2 ER Stress Signalling in PTI

**FIGURE 3:**
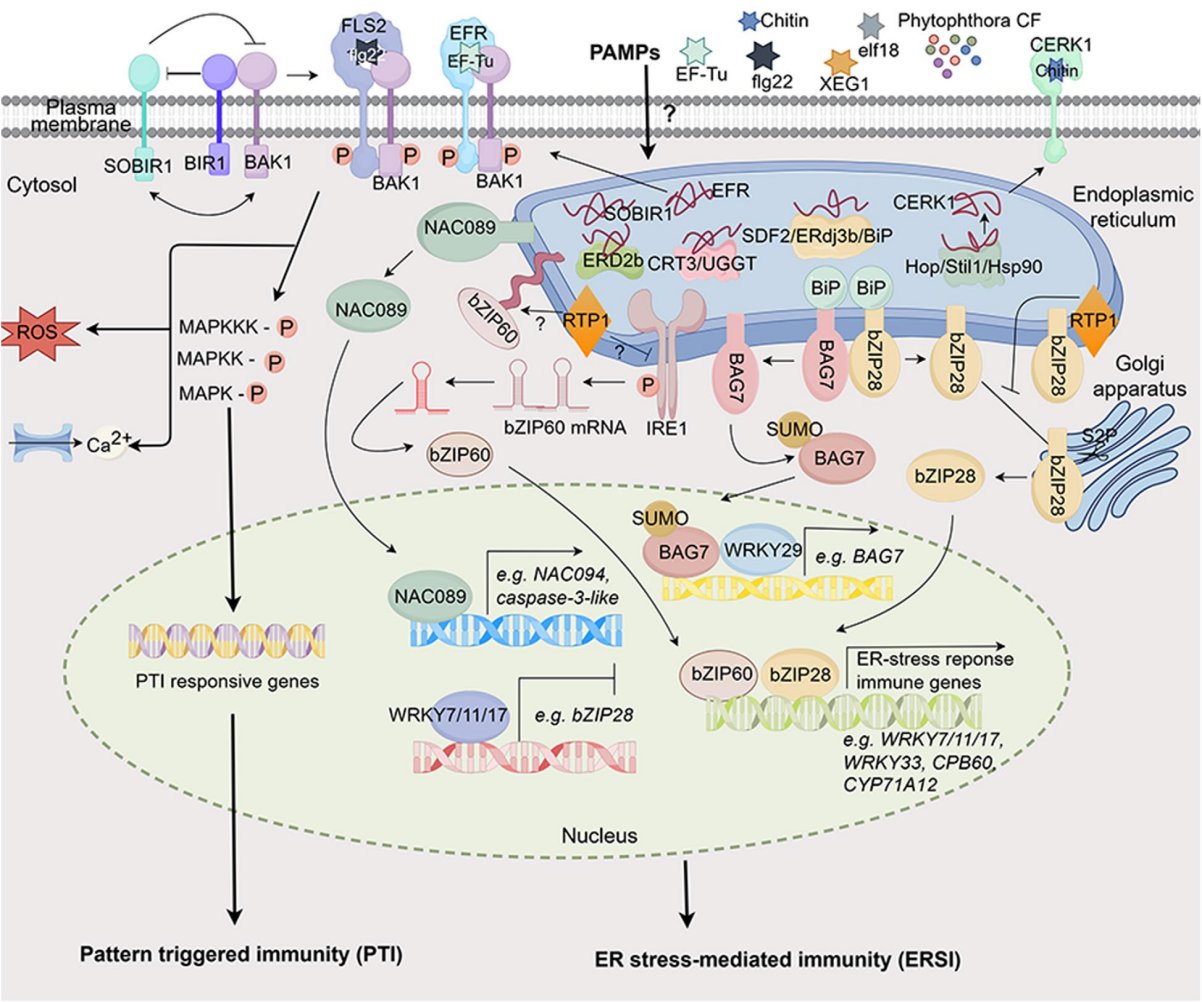
Integration of PTI and ER stress‐mediated immunity in plant defence. PAMP perception activates both PTI and ER stress signalling. Recognition of PAMPs such as *Phytophthora* culture (CF), chitin, flg22, EF‐Tu, XEG1 and elf18 promotes the release, processing, and nuclear translocation of ER stress regulators bZIP28, BAG7 and NAC089, which in turn activate ER stress‐induced defence (ERSI) genes, including *WRKY7/11/17* and *NAC094*. Proper folding and trafficking of PRRs such as EFR, SOBIR1 and CERK1 depend on ERQC components, including CRT3, UGGT, ERD2b, SDF2, ERdj3b, BiP, Hop/Sti1 and Hsp90. The susceptibility factor RTP1 attenuates both PTI and the UPR by stabilising ER‐anchored bZIP28 through direct protein interaction and unspliced bZIP60 via an unknown mechanism, while also suppressing IRE1‐dependent bZIP60 mRNA splicing. WRKY7, WRKY11 and WRKY17 further repress *bZIP28* expression by binding its promoter.

The activation of PTI upon PAMP perception is intricately linked to the induction of ER stress signalling, forming a rapid early layer of intracellular response that potentiates defence (Figure 3). The culture filtrate (CF) of *P. capsici*, which contains a variety of PAMPs, induces the expression of ER stress‐related genes, such as *bZIP60*, *BiP3*, *BiP1/2*, *BI‐1*, *PDI*, *ERdj3*, *CNX* and *NAC089*, in a manner akin to Tm, a chemical ER stress inducer that inhibits protein N‐glycosylation (Ai et al. 2021). Furthermore, treatment with bacterial PAMP flg22 or the *Phytophthora* PAMP XEG1 also modulates ER stress gene expression, albeit with distinct patterns compared to those triggered by *P. capsici* CF, suggesting that different PAMPs elicit specific ER stress responses (Arraño‐Salinas et al. 2018; Ai et al. 2021). Tm treatment enhances the expression of PTI marker genes (*FRK1*, *NHL10* and *WRKY29*) and activates MAPK phosphorylation, a key event in defence signalling (Ai et al. 2021). These findings underscore the intricate interplay between ER stress and PTI pathways (Figure 3).

PAMPs induce the release, cleavage, and nuclear translocation of key UPR membrane‐tethered transcription factors (MTFs), such as bZIP28, BAG7, and NAC089, thereby activating downstream UPR signalling involved in ER stress‐mediated immunity (ERSI) (Figure 3) (Zhou et al. 2021; Ai et al. 2021; Arraño‐Salinas et al. 2018). The bZIP28 pathway plays a pivotal role in PTI responses to PAMPs like flg22 and *Phytophthora* CF (Arraño‐Salinas et al. 2018; Zhou et al. 2021). Upon PAMP recognition, the bZIP28‐BAG7 complex—normally retained in the ER—translocates to the nucleus to regulate ER stress‐mediated immunity (ERSI). WRKY7, WRKY11 and WRKY17 transcription factors bind to the bZIP28 promoter to repress its expression, thereby suppressing PTI by downregulating bZIP28 after flg22 activation (Figure 3) (Journot‐Catalino et al. 2006; Kim et al. 2006; Arraño‐Salinas et al. 2018). RTP1, a susceptibility factor, negatively regulates both PTI defence and the UPR by suppressing IRE1‐dependent bZIP60 splicing and stabilising inactive bZIP28 via direct interaction (Figure 3) (Qiang et al. 2021). In response to microbial PAMPs (*Phytophthora* CF, flg22, or *Pst* DC3000 hrcC^−^), NAC089 translocates from the ER to the nucleus—where it initiates ER stress responses and enhances caspase‐3‐like activity—in a manner dependent on brassinosteroid insensitive 1 (BRI1)‐associated receptor kinase 1 (BAK1), a PTI co‐receptor for both flagellin‐sensitive 2 (FLS2) and elongation factor‐Tu (EF‐Tu) receptor (EFR) (Ai et al. 2021; Gómez‐Gómez and Boller 2000; Zipfel et al. 2006; Chinchilla et al. 2007; Heese et al. 2007). NAC089 induces downstream gene *NAC094* and positively contributes to host resistance against *P. capsici* and *Pst* DC3000 (Ai et al. 2021).

Molecular chaperones are indispensable for PTI, ensuring the folding, stability, and trafficking of plasma membrane‐localised PRRs (Figure 3). Loss of ERQC components such as *CRT3*, *UGGT*, *ERD2b*, *SDF2* and *ERdj3B* disrupts EFR maturation and increases susceptibility (Li et al. 2009; Semenza et al. 1990; Lewis and Pelham 1992; Nekrasov et al. 2009), underscoring the roles of CNX/CRT3 and SDF2–ERdj3B–BiP systems in receptor biogenesis. Similar requirements apply to XA21 and SOBIR1, whose accumulation and function also depend on ERQC machinery (Park et al. 2013; Song et al. 1995; Pruitt et al. 2015; Gao et al. 2009; Thomas et al. 2014; Sun et al. 2014). The rice chitin receptor CERK1 further illustrates this principle: its ER‐to‐PM transport relies on Hsp90 and Hop/Sti1 (Chen et al. 2010), which also assemble with OsRac1 into “defensomes” at the plasma membrane to mediate immune signalling (Chen et al. 2010). Collectively, these findings establish ERQC chaperones as critical determinants of PRR maturation and as active components of immune signalling complexes.

### 3.3 ER Stress Signalling in ETI

**FIGURE 4:**
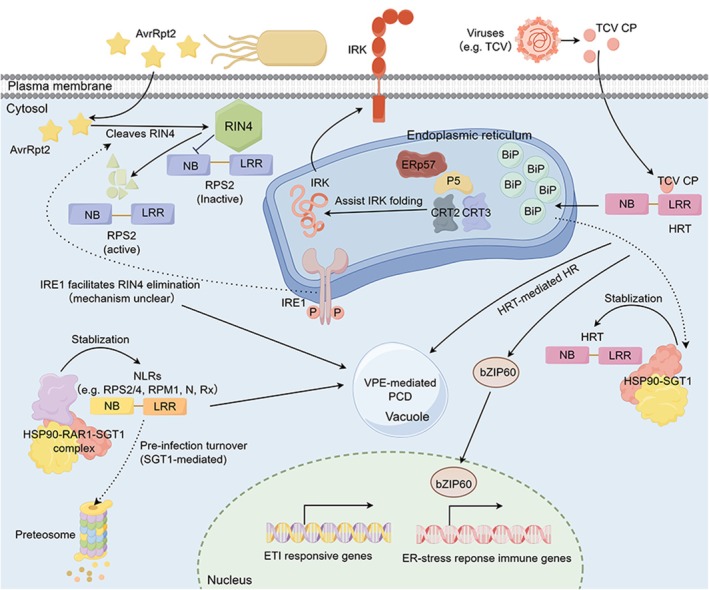
Integration of ETI and ER stress‐mediated immunity in plant defence. *Turnip crinkle virus* (TCV) coat protein (CP) is recognised by the NLR receptor HRT, triggering ER stress and activating UPR core components BiP4/5 and bZIP60. BiP4/5 positively regulates expression of HSP90 and SGT1, which synergistically maintain HRT protein stability to ensure execution of hypersensitive response (HR). The bacterial effector AvrRpt2 cleaves the guardee protein RIN4, releasing the NLR receptor RPS2 to trigger ETI. IRE1 facilitates efficient RIN4 degradation by AvrRpt2, thereby potentiating RPS2 activation. Other NLRs (e.g., RPS2/RPS4, RPM1, N and Rx) require the cytosolic HSP90‐RAR1‐SGT1 complex for stabilisation. SGT1b counteracts RAR1 to promote R protein turnover before infection, but supports their accumulation during immune activation. In the ER lumen, the receptor‐like kinase IRK depends on CRT2, CRT3, ERp57 and P5 for correct folding and maturation before mediating N‐dependent HR‐PCD against TMV.

Chaperones also ensure the proper folding and stability of certain R proteins involved in ETI, maintaining them in a functional state at the appropriate cellular locations (Figure 4). The cytosolic chaperone complex SGT1–RAR1–HSP90 is critical for the stability and signalling capacity of multiple NLR proteins—key receptors in plant ETI. Examples include N, Rx, RPM1, RPS2 and RPS4, which confer resistance against various viral and bacterial pathogens. HSP90, in conjunction with the co‐chaperones RAR1 and SGT1, directly interacts with these NLRs to regulate their accumulation and activation (Hubert et al. 2003; Takahashi et al. 2003; Zhang et al. 2004; Liu et al. 2004; Whitham et al. 1994; Grant et al. 1995; Lu et al. 2003). In *Arabidopsis*, SGT1b plays a dual role in immune regulation (Holt et al. 2005). Prior to infection, SGT1b counteracts RAR1 to promote the degradation of R proteins such as RPS5, RPM1, and RPP4/8/31 (Holt et al. 2005). However, during infection, SGT1b interacts with RAR1 and HSP90 to facilitate the accumulation of R proteins, thereby helping maintain their stability (Holt et al. 2005). Thus, the coordinated actions of RAR1, SGT1, and HSP90 finely control both the pre‐activation turnover and functional accumulation of R proteins. Chaperones are also vital for the proper folding and maturation of immune‐related proteins during ETI. One such key component is the leucine‐rich repeat (LRR) receptor‐like kinase IRK, which localises to the plasma membrane and is indispensable for the N‐mediated hypersensitive response and programmed cell death (HR‐PCD) against TMV (Caplan et al. 2009). Upon ETI activation in *N. benthamiana*, the ER‐resident calreticulins CRT2 and CRT3 are transcriptionally upregulated and are essential for IRK protein accumulation (Figure 4) (Caplan et al. 2009). Functional silencing of *NbCRT2* and *NbCRT3*, along with the protein disulfide isomerases *NbERp57* and *NbP5*, partially compromises the N‐mediated immune response to TMV (Caplan et al. 2009). It is proposed that CRTs form multi‐chaperone complexes with PDIs to ensure the correct conformation and function of IRK (Caplan et al. 2009).

The ER stress response significantly contributes to HR, a localised form of cell death mediated by R proteins that restricts pathogen spread. In *A. thaliana* , the NLR protein HRT recognises the coat protein (CP) of TCV, triggering HR and conferring viral resistance to TCV (Figure 4) (Moon et al. 2016). Co‐expression of HRT and TCV CP in *N. benthamiana* induces robust activation of ER stress‐responsive genes and BiP chaperone accumulation (Moon et al. 2016). Overexpression of *BiP* in soybean and tobacco amplifies HR, whereas *BiP* silencing in tobacco weakens HR (Carvalho et al. 2014). Transcriptome analysis following *BiP* overexpression shows a significant upregulation of hypersensitive PCD‐related genes involved in nonhost resistance (Carvalho et al. 2014). Specifically, BiP4/5, along with HSP90 and SGT1, modulate HRT‐mediated HR by enhancing HRT expression at both mRNA and protein levels (Figure 4) (Moon et al. 2016). Silencing *BiP4/5*, *SGT1* and *HSP90* alters ER stress responses and modifies defence gene expression in response to HRT or TCV CP (Moon et al. 2016). Furthermore, Tm treatment impairs HRT‐mediated HR, as ER stress and N‐glycosylation affect HRT accumulation and stability, leading to reduced cell death activation (Moon et al. 2016). Collectively, these studies demonstrate that an optimally functioning UPR is required for the full execution of ETI‐associated HR.

Further emphasising the intimate link between UPR and ETI, recent work has uncovered a direct role for the ER stress sensor IRE1 in regulating the AvrRpt2‐RIN4‐RPS2 immune axis (Figure 4) (Chakraborty et al. 2020). RIN4 functions as a molecular ‘guardee’ that maintains the NLR protein RPS2 in an inactive state by forming a stable complex, thereby preventing autoimmunity in the absence of pathogens. The *Pseudomonas syringae* effector AvrRpt2, a cysteine protease, specifically cleaves RIN4, leading to its rapid degradation. This degradation event relieves RIN4‐mediated suppression of RPS2, triggering ETI and HR. Remarkably, IRE1 functions as an upstream facilitator of this process: under ER stress conditions, IRE1 loss‐of‐function mutants exhibit delayed RIN4 degradation upon AvrRpt2 delivery and enhanced susceptibility to avirulent *Pst* DC3000 (AvrRpt2) (Chakraborty et al. 2020). Thus, IRE1 specifically potentiates the AvrRpt2‐RIN4‐RPS2 axis by promoting efficient RIN4 elimination, thereby ensuring robust ETI activation.

Together, this intimate integration of ER homeostasis with ETI underscores how plants have co‐opted the UPR as an active participant in executing robust immune responses.

### 3.4 ER Stress Signalling in Phytohormone Pathways for Systemic Immunity

Emerging evidence positions ER stress signalling as a key modulator within the hormone‐driven systemic defences, engaging in bidirectional crosstalk with both SA and JA pathways that fine‐tunes the output of the plant immune response.

#### 3.4.1 SA as an ER Stress Signalling Regulator in Plants

**FIGURE 5:**
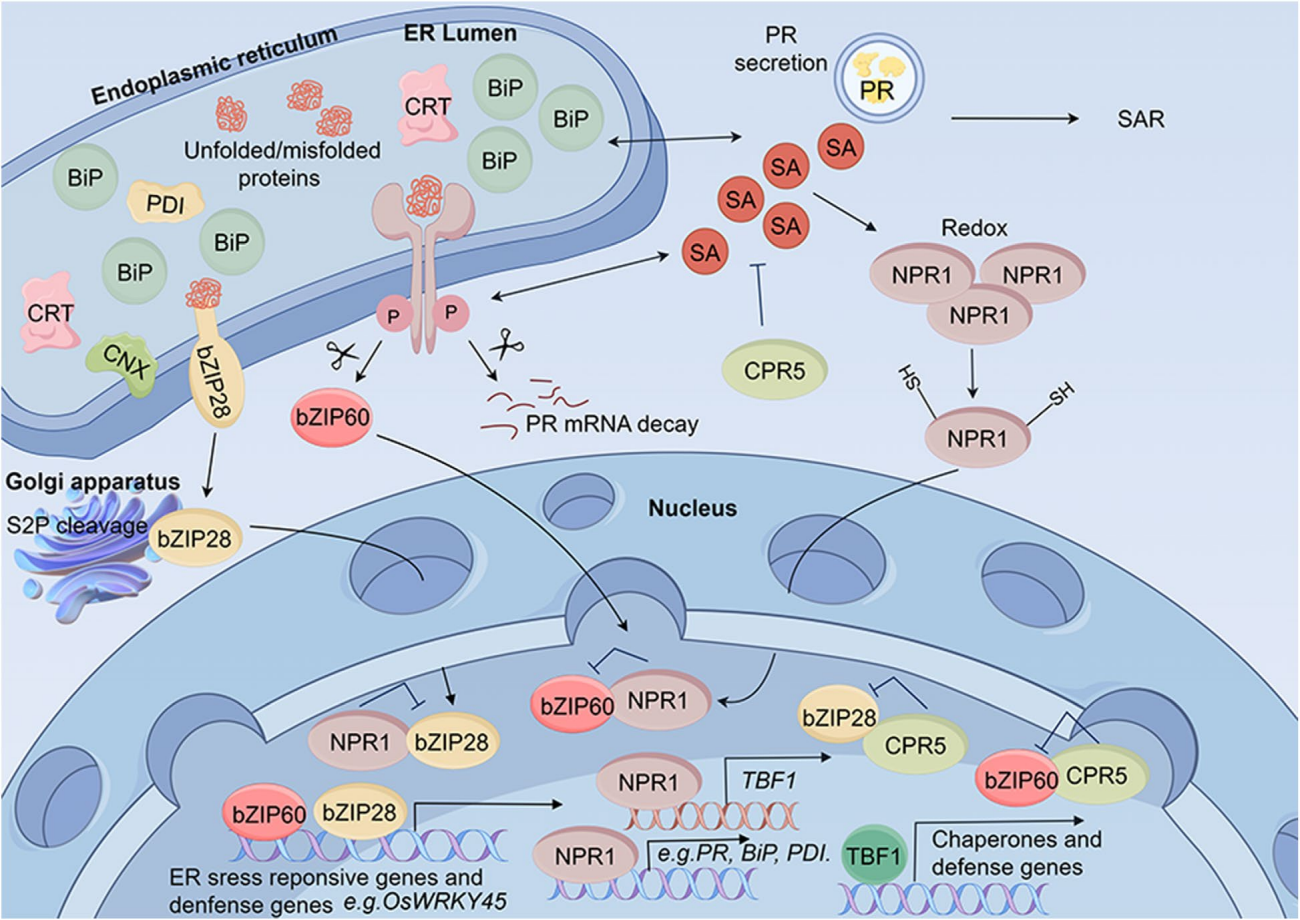
SA as an ER stress signalling regulator in plants. Salicylic acid (SA) activates a subset of unfolded protein response (UPR) genes and defence‐related genes, including molecular chaperones, *OsWRKY45* and *TBF1*, primarily through the bZIP28 and IRE1‐bZIP60 branches. CPR5 counteracts SA accumulation and directly interacts with bZIP28 and bZIP60 to suppress their transcriptional activity. ER stress and SA‐induced redox changes promote NPR1 monomerisation and nuclear import, where NPR1 associates with bZIP28 and bZIP60, attenuating UPR amplitude while activating pathogenesis‐related (PR) genes. NPR1 also coordinates expression of ER chaperones (*BiP*, *CNX*, *CRT* and *PDI*) that are essential for PR protein folding and secretion. TBF1 links NPR1 to UPR gene activation by binding chaperone gene promoters, although its role appears context‐dependent. IRE1 fine‐tunes immune output through regulated RIDD‐mediated selective decay of secretory protein mRNAs, including subsets of PR transcripts. Reciprocally, the IRE1‐bZIP60 branch is essential for SA‐dependent systemic acquired resistance (SAR), and overexpression of *BiP* or *CRT2* potentiates SA signalling. Together, SA and the UPR form a bidirectional regulatory circuit that constitutes a molecular backbone for SAR.

The induction of SAR depends on the accumulation of SA, which activates gene expression through the master regulator non‐expresser of pathogenesis‐related genes 1 (NPR1). Exogenous SA also triggers SAR and the expression of *NPR1* and *PR* genes, even without the presence of pathogens (Ward et al. 1991). Furthermore, SA signalling is intimately linked to the UPR, forming a complex bidirectional regulatory circuit (Figure 5).

Exogenous SA activates the UPR at high concentrations (Pajerowska‐Mukhtar et al. 2012; Nagashima et al. 2014). The transcriptome induced by SA partially overlaps with genes activated in the UPR (Krinke et al. 2007). In *Arabidopsis*, SA application significantly alters the expression of genes encoding ER‐resident proteins involved in protein folding and secretion, such as *BiP2* and *BiP3* (Wang et al. 2005; Nagashima et al. 2014). Exogenous SA activates UPR genes via two primary signalling pathways: bZIP28 and IRE1‐bZIP60 (Figure 5) (Nagashima et al. 2014). Pathogen infection also induces UPR genes in a SA‐dependent manner (Chandran et al. 2009). However, the role of endogenous SA in activating the UPR remains controversial. Infection by *D. gigantea* significantly increased SA concentrations in wild‐type (WT) plants but not in the *bzip28 bzip60* mutants, which exhibited increased susceptibility to the pathogen (Samperna et al. 2021). In WT plants, transcript levels of isochorismate synthase 1 (*ICS1*), an essential enzyme for SA biosynthesis, increased after pathogen exposure, while the *bzip28 bzip60* mutants did not show this response (Samperna et al. 2021). These results suggest that an intact UPR is crucial for boosting endogenous SA levels, which may further enhance UPR via a positive feedback loop. However, the SA‐induction‐deficient 2 (*sid2*) mutant, which lacks ICS1 and cannot produce SA, exhibits ER‐stress responses similar to WT plants, as Tm treatment does not significantly alter UPR marker gene expression compared to WT (Lai et al. 2018). Additionally, Tm treatment did not alter free or conjugated SA levels, nor did it activate SA‐dependent signalling—such as the transcription of *CBP60g*, *SID2* and *PR1*—in either WT or *sid2* plants (Lai et al. 2018). In contrast, Wang et al. reported that Tm treatment caused a four‐fold increase in SA content in *Arabidopsis* plants (Guo et al. 2018). These conflicting results may be due to differences in seedling age, reagent concentrations, or Tm treatment duration.

More compelling evidence for this integrative role comes from studies on key SA signalling components. NPR1, the master SA receptor, directly participates in UPR by physically interacting with bZIP28 and bZIP60 (Lai et al. 2018). Upon ER stress, SA accumulation and a concomitant cytosolic redox shift promote NPR1 monomerisation and nuclear translocation, where it suppresses the transcriptional activity of bZIP28 and bZIP60, thereby attenuating UPR amplitude and fine‐tuning cytoprotective output (Figure 5) (Lai et al. 2018). This NPR1‐mediated attenuation intersects with the TBF1 pathway: *npr1* mutants display hyper‐induction of TBF1‐dependent chaperone genes such as *CNX1*, *BiP2* and *PDIL* (Lai et al. 2018). TBF1, an HSF‐like transcription factor, can bind the TL1 cis‐element in defence‐related and ER chaperone promoters, thereby facilitating NPR1‐dependent UPR gene activation and PR1 secretion during defence (Pajerowska‐Mukhtar et al. 2012). However, TBF1's role in the UPR appears context‐sensitive. While some studies report that *tbf1* mutants exhibit hypersensitivity to Tm and *TBF1*‐overexpression enhances tolerance (Pajerowska‐Mukhtar et al. 2012; Hossain et al. 2016), others observed no difference in Tm sensitivity or UPR gene induction (Nagashima et al. 2014), and one showed only transient hyper‐induction of UPR genes at an early time point (12 h post‐Tm) (Lai et al. 2018). These discrepancies underscore the need for standardised assays to clarify the NPR1–TBF1–UPR axis. Adding another layer of complexity, CPR5, which functions downstream of pathogen recognition and upstream of NPR1‐dependent SA signalling to regulate cell cycle‐related ETI‐induced PCD (Bowling et al. 1997; Wang et al. 2014), also acts as a positive growth modulator under both physiological and ER‐stress conditions, counteracting SA‐dependent growth inhibition via UPR modulation (Figure 5). The *cpr5* mutant, which exhibits constitutive expression of PR genes and elevated SA levels, demonstrates reduced sensitivity to chronic ER stress and enhanced basal UPR (Meng et al. 2017; Bowling et al. 1997). Notably, the enhanced basal UPR in *cpr5* is SA‐independent but is crucial for resistance to chronic ER stress (Meng et al. 2017). Mechanistically, CPR5 physically interacts with and suppresses bZIP28 and bZIP60, thereby contributing to SA‐mediated growth inhibition and managing the trade‐off between plant growth and stress responses (Meng et al. 2017).

Finally, the UPR also feeds back to influence SA biology (Figure 5). The IRE1‐bZIP60 branch orchestrates early, SA‐gated SAR against bacterial pathogens. While *ire1a* mutants exhibit heightened susceptibility, impaired SAR, and defective bZIP60 splicing upon pathogen challenge or SA application, *bzip60* mutants only weakly affect SA‐responsive gene induction and maintain normal PR1 secretion, indicating that *IRE1a* also modulates other clients independently of *bZIP60* (Moreno et al. 2012). In rice, IRE1 down‐regulates a subset of *PR* genes via RIDD‐mediated mRNA decay to alleviate ER stress during the immune response (Hayashi et al. 2012; Verchot and Pajerowska‐Mukhtar 2021). OsWRKY45, a SA‐inducible TF, is transcriptionally activated by OsIRE1‐OsbZIP50 (the homologue of *Arabidopsis* bZIP60) during ER stress and counterbalances reduced PR expression by amplifying SA‐dependent defences (Hayashi et al. 2012). Notably, *BiP* overexpression potentiates SA signalling—elevating SA levels, upregulating *PR1*/*PR5*, and accelerating HR‐PCD, thereby priming plants for amplified defence upon pathogen challenge (Carvalho et al. 2014). Similarly, *CRT2* overexpression increases SA accumulation and constitutively activates SAR markers PR1/PR2/PR5, yet paradoxically reduces resistance to *Pst* DC3000 (Qiu et al. 2012). CRT2 thus functions dually: its Ca^2+^‐binding C‐terminus promotes SA biosynthesis, while its N‐terminal chaperone module attenuates SA‐driven immunity to prevent runaway defence (Qiu et al. 2012).

Collectively, exogenous SA triggers the UPR, while disruption of endogenous SA alters UPR responses, underscoring the role of SA in tuning ER stress. Reciprocally, ER stress enhances SA biosynthesis and selectively tunes SA‐responsive gene expression through the SA master regulators NPR1, TBF1 and CPR5. Consequently, the SA‐ER‐stress nexus constitutes an essential molecular backbone for systemic acquired resistance against pathogens.

#### 3.4.2 JA as an ER Stress Signalling Regulator in Plants

JA promotes the rapid production of antifungal proteins such as defensins and protease inhibitors and enhances defence enzyme activity in response to infection or wounding (Wasternack and Hause 2013; Hu et al. 2009). Emerging evidence links JA to UPR signalling, positioning JA as an additional regulator of ER‐stress‐mediated defence. JA controls UPR gene expression across species. In tobacco, *A. alternata* infection upregulates key UPR‐related genes, including *BiP*, *PDI*, *CNX1‐like*, *CRT*, *IRE1* and *bZIP60*. This response is abolished in JA‐deficient or JA‐insensitive lines but is mimicked by exogenous methyl jasmonate (MeJA) treatment (Xu et al. 2019). JA‐responsive genes *AOS* and *LOX3* are not significantly altered when the IRE1‐bZIP60 pathway is disrupted (Xu et al. 2019), suggesting that this module functions downstream of JA biosynthesis during the response to fungal attack. Tomato data mirror this hierarchy: MeJA elevates *BiP*, *IRE1* and *bZIP60* transcripts, while the JA‐insensitive *jai1* mutant down‐regulates *IRE1a/b* yet paradoxically upregulates *BI1* and *bZIP60* (Czékus et al. 2020). JA also modulates H_2_O_2_ levels, proteasome activity, and lipid peroxidation under Tm‐induced ER stress (Czékus et al. 2020). In soybean, *BiP* overexpression lowers endogenous JA and represses multiple JA‐biosynthetic genes (Carvalho et al. 2014; Coutinho et al. 2019). In *Medicago truncatula* , JA signalling induces triterpene saponin (TS) biosynthesis, a defence related process, via the transcription factors TSAR1 and TSAR2, whereas bZIP60 and bZIP17, whose expression and processing are modulated by JA and TS levels, repress TSAR1 and TSAR2 activity, thereby fine‐tuning TS production and JA‐mediated defence (Ribeiro et al. 2022).

Collectively, JA acts as an upstream signal capable of triggering UPR gene expression, while the engaged UPR machinery, in turn, feeds back to modulate JA pathway outputs. Although mechanistic details remain incomplete, JA's central regulatory role is now unequivocal.

## 4 Functionality of the ER Stress Response During Pathogen Infection

**FIGURE 6:**
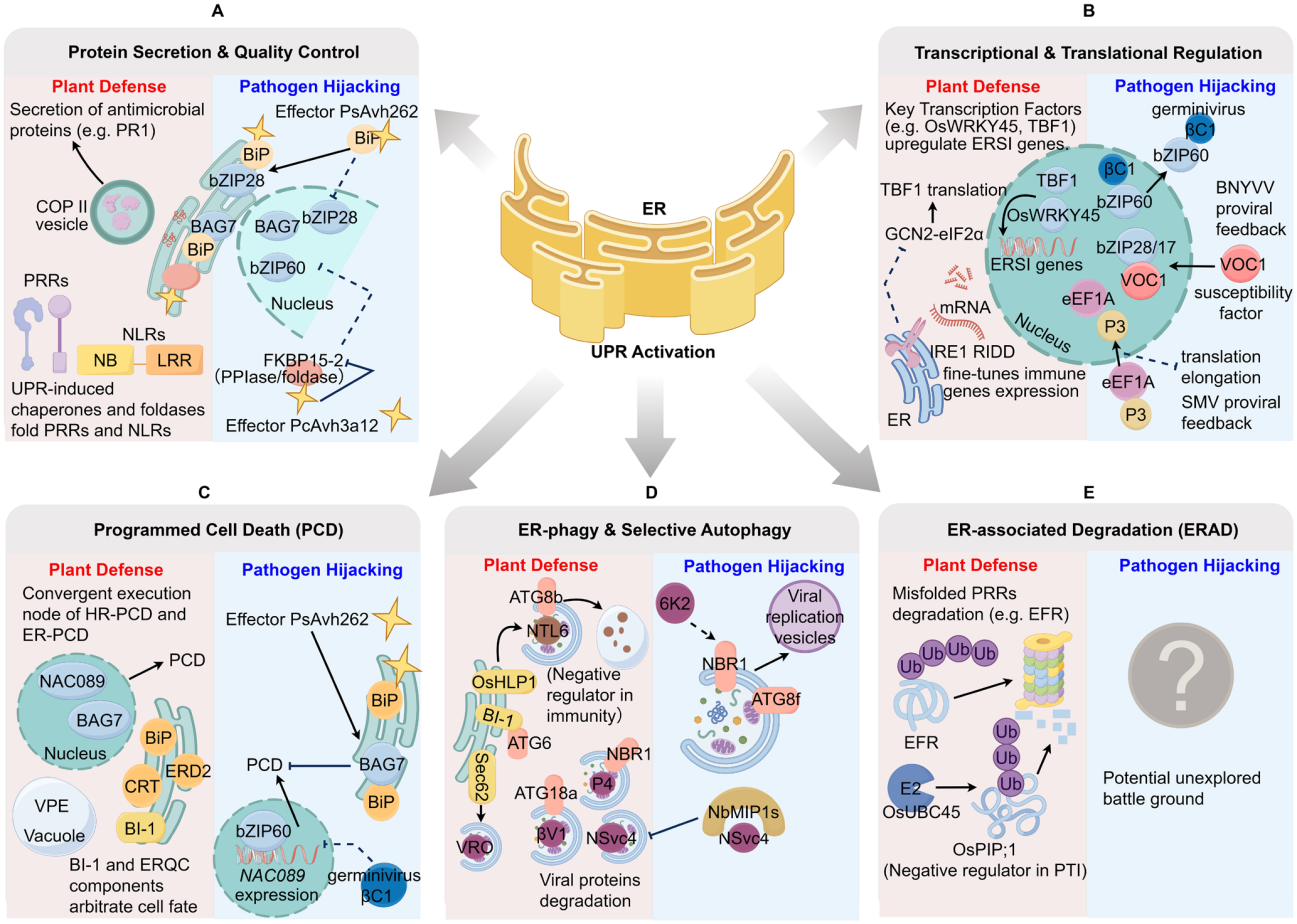
UPR downstream mechanisms in plant immunity: Defence and pathogen hijacking. This diagram integrates five functional modules downstream of UPR activation, depicting both plant defence roles (blue) and pathogen hijacking strategies (orange), arranged around a central ER icon. (A) Protein secretion and quality control. UPR‐induced chaperones facilitate PRR and NLR folding and promote secretion of antimicrobial proteins (e.g., PR1). PsAvh262 stabilises BiP and traps BAG7 and bZIP28 in the ER, blocking their transcriptional activation, while PcAvr3a12 inhibits the foldase FKBP15‐2 to compromise ER stress‐mediated immunity (ERSI). (B) Transcriptional and translational regulation. UPR‐activated immune‐related transcription factors (OsWRKY45, TBF1) upregulate ERSI genes. IRE1 RIDD fine‐tunes immune gene expression (e.g., *PR‐4*, *EDS1 and WRKY33*) by reducing their mRNA aboundance. IRE1 also maintains translational equilibrium by modulating the GCN2‐eIF2α axis that enhancing TBF1 translation for immune signal amplification. *Geminivirus* βC1 promotes *bZIP60* expression but exports bZIP60 from the nucleus; BNYVV utilises the host susceptibility factor VOC1 to enhance bZIP28/17 activity in a proviral feedback loop; and SMV P3 targets eEF1A, causing its nuclear relocalisation and aberrant translation elongation, thereby inducing non‐canonical ER stress. (C) Programmed cell death (PCD). HR‐PCD and ER‐PCD converge on VPE as a common executioner, with NAC089, BAG7, BI‐1 and ERQC components calibrating cell fate. PsAvh262 retains BAG7 in the ER to block PCD, and βC1 suppresses *NAC089* expression via bZIP60 nuclear export. (D) ER‐phagy and selective autophagy. ER‐phagy receptors (Sec62, ATG18a and NBR1) mediate degradation of viral proteins (e.g., CaMV P4, RSV NSvc4, *geminivirus* βV1) or viral replication organelles (VROs). ER‐phagy receptor OsHLP1 specifically cooperates with OsATG8b to degrade the immune suppressor OsNTL6, thereby revealing ER‐phagy as an active arm of immune signalling. BI‐1 recruits ATG6 to initiate ER‐phagy. TuMV 6K2 upregulates NBR1 to recruit ATG8, protecting viral replication vesicles, and NbMIP1s shields RSV NSvc4 from autophagic clearance. (E) ER‐associated degradation (ERAD). ERAD removes misfolded PRRs (e.g., EFR) and targets negative regulators such as OsPIP2; 1 (via OsUBC45) to enhance PTI. Pathogen hijacking of ERAD has not yet been reported, leaving this interface a potentially unexplored front in plant‐pathogen coevolution.

The preceding sections position the UPR not merely as a stress mitigator but as an active orchestrator of defence. To synthesise these insights, this section examines the core functional modules through which the UPR executes its immune‐regulatory roles: (i) quality control of immune receptors and secreted effectors, (ii) transcriptional and translational reprogramming of defence genes, (iii) calibrated regulation of PCD, and (iv) induction of autophagy (Figure 6). By dissecting these modules, we aim to provide a unified framework for understanding how the UPR translates pathogen perception into a coherent, multi‐layered immune response.

### 4.1 UPR‐Driven Quality Control in Immune Protein Biogenesis and Secretion

The efficacy of plant innate immunity hinges on the rapid production and deployment of a diverse array of immune receptors, signalling intermediates, and antimicrobial proteins. The UPR fulfils a foundational role in this process by enforcing stringent quality control (QC) on the proteome of the secretory pathway, ensuring that defence components are correctly folded, assembled, and trafficked to their sites of action (Figure 6A) (Simoni et al. 2022; Park et al. 2013).

This QC function operates at multiple layers of immunity. As detailed in Sections 3.2 and 3.3, UPR‐boosted chaperones and foldases secure the biogenesis, stability, and function of both cell‐surface PRRs and intracellular NLRs, thereby enabling rapid pathogen detection and downstream immune responses. The fundamental nature of this support is further underscored by the function of the potato ER‐membrane protein StDMP2, which enhances late‐blight resistance by amplifying *P. infestans* ‐triggered UPR and specifically promoting the maturation of multiple PRRs, including StPERU, StFLS2, and StCERK1, as well as the ETI regulator StNDR1 (Bi et al. 2025).

The UPR is indispensable for the secretion of antimicrobial proteins that constitute the plant's first defensive barrier and for the proactive establishment of defence readiness. Within minutes of pathogen perception, chaperones such as BiP are transcriptionally upregulated, thereby priming ER folding capacity before defence proteins enter COPII vesicles destined for infection sites and before a surge of nascent defence proteins arrives (Simoni et al. 2022; Janssens et al. 2014; Jelitto‐Van Dooren et al. 1999). Upon attack, PR proteins and other immune cargoes traverse the ER and their secretion is strictly gated by the UPR. Comparative proteomics of the *bzip28 bzip60* double mutant after fungal challenge shows a selective loss of 42 defence proteins, 27 of which are annotated as secreted or ER‐resident, underscoring the ER as a bottleneck for immune cargo maturation (Samperna et al. 2021). The pivotal role of this UPR‐driven secretory burst is highlighted by the fact that IRE1a is essential for PR secretion, with secretion collapsing in *ire1a* mutants and being virtually abolished in *ire1a ire1b* double mutants (Moreno et al. 2012). Successful secretion further requires the ER luminal chaperone BiP2 and its co‐chaperone DAD1, since the loss of either one blocks NPR1‐dependent PR1 release (Wang et al. 2005).

Thus, by securing the fidelity and efficient secretion of the immune proteome, UPR‐mediated quality control establishes the essential molecular substrate upon which all subsequent defence responses are built, connecting pathogen detection to the effective mobilisation of antimicrobial outputs.

### 4.2 Regulation of Transcription and Translation

UPR‐mediated coupling of transcription and translation synchronises the rapid production and turnover of defence molecules, thereby strengthening resistance while preserving cellular homeostasis during pathogen attack (Figure 6B).

The UPR deploys transcription factors to directly control immune gene expression. For example, the rice transcription factor WRKY45 is directly induced by the OsIRE1–OsbZIP50 pathway under ER stress and subsequently activates SA‐responsive defence genes, consolidating its immune‐potentiating role (Hayashi et al. 2012). Likewise, in *Arabidopsis*, the transcription factor TBF1, a key factor involved in the UPR and acting downstream of SA signalling as discussed in Section 3.4.1, is activated upon pathogen challenge to repress growth‐related genes while inducing ER‐resident chaperones and secretion‐pathway components, thereby reinforcing ER capacity for defence output (Pajerowska‐Mukhtar et al. 2012). In addition to its transcriptional control through regulated IRE1‐dependent splicing (RIDS), IRE1 fine‐tunes immune outputs post‐transcriptionally via regulated RIDD, selectively degrading mRNAs of immune‐associated genes such as *PRX34*, *EDS1*, *WRKY33*, *WRKY53*, *WRKY70*, *MLO4* and *PR‐4* to adjust protein synthesis during pathogen‐induced ER stress (Mishiba et al. 2013). Thus, through both the transcriptional induction of defence‐related genes and the selective decay of specific mRNAs, the UPR dynamically reshapes the transcriptomic landscape to fine‐tune immune signalling outputs.

Beyond its role in mRNA stability control, emerging evidence positions IRE1 as a crucial guardian of translational homeostasis. Notably, the loss of *IRE1* leads to hyperactivation of GCN2 and consequent excessive phosphorylation of the translation initiation factor eIF2α, a change that is associated with severe global translational suppression under ER stress (Yoo et al. 2024). This reveals a critical crosstalk wherein the canonical UPR (via IRE1) maintains translational equilibrium by modulating the GCN2‐eIF2α axis. The GCN2‐eIF2α module represents a central, yet plant‐distinct, pathway for translational control during immunity. Unlike the PERK‐eIF2α branch of the mammalian UPR, plant GCN2 is not a dedicated ER stress sensor but a multifunctional integrator activated by diverse immune signals, including chemical ER inducers (e.g., dithiothreitol but not Tm), PAMPs (e.g., chitin), and defence hormones (SA and JA) (Wynn et al. 2025; Kamauchi et al. 2005; Izquierdo et al. 2018; Berrocal‐Lobo et al. 2020). Although GCN2 shares the conserved function of phosphorylating eIF2α, this modification in plants often facilitates selective translational reprogramming rather than the wholesale protein synthesis shutdown characteristic of the mammalian PERK pathway (Immanuel et al. 2012; Izquierdo et al. 2018). A major output of this reprogramming is the translational de‐repression of TBF1. The *TBF1* mRNA contains upstream open reading frames (uORFs) that render its translation sensitive to eIF2α phosphorylation—a regulatory mechanism analogous to that governing *ATF4* in the mammalian PERK‐eIF2α arm of the UPR (Hinnebusch 2005; Pajerowska‐Mukhtar et al. 2012). Upon immune activation, GCN2‐mediated eIF2α phosphorylation facilitates ribosomal bypass of the inhibitory uORFs, enabling a rapid burst of TBF1 protein synthesis. This translationally controlled TBF1 pulse amplifies the immune signal: the synthesised TBF1 protein enters the nucleus to drive the transcription of ER chaperone and defence genes and to fine‐tune stomatal immunity by modulating the ABA signalling pathway (Liu et al. 2019; Pajerowska‐Mukhtar et al. 2012). Thus, TBF1 functions within a self‐reinforcing loop: it is transcriptionally induced to bolster ER function, and its translation is further potentiated by the GCN2‐eIF2α axis under stress, amplifying a comprehensive defence response that integrates systemic proteostatic adaptation with localised physiological defences.

Thus, the UPR coordinates transcriptional and translational programs to ensure that the immune outputs are robust yet precisely regulated, optimising the trade‐off between effective immunity and cellular homeostasis.

### 4.3 Priming of PCD

ER‐stress components act as pivotal regulators of pathogen‐triggered PCD, guiding cell‐fate decisions during microbial attack (Figure 6C). ER‐stress‐induced PCD and HR‐PCD appear to converge on shared executioners such as VPE‐mediated vacuolar collapse, yet they rely on distinct upstream cues to counter different layers of pathogen challenge. This ‘split‐yet‐integrated’ strategy allows plants to deploy spatially and mechanistically customised cell‐death programs against diverse invaders.

#### 4.3.1 ER‐PCD and HR‐PCD

Although PCD is well‐established in plants, a strict categorisation of its different types remains less defined than in animal systems (Van Doorn 2011). No homologues of Bcl‐2, BAX/BAK or caspases are present; instead, plants rely on functional equivalents such as Vacuolar Processing Enzymes (VPE, caspase‐1‐like), cathepsin B (caspase‐3‐like), and Bax inhibitor‐1 (Oh et al. 2003; Xu and Reed 1998; Cai et al. 2018; Hatsugai et al. 2006). Two major immune‐related PCD types are HR‐PCD, initiated by NLR receptors upon pathogen detection (Lam 2004; Jones and Dangl 2006), and ER stress‐triggered PCD (ER‐PCD), activated when unresolved ER stress shifts signalling from pro‐survival to pro‐death (Watanabe and Lam 2008a; Mishiba et al. 2013; Yang et al. 2016; Shore et al. 2011; Bao et al. 2018; Yang, Wang, et al. 2014). Although initiated by distinct sensors, both converge on shared execution modules.

VPEs execute both HR‐ and ER‐PCD by cleaving vacuolar membrane proteins, inducing lytic collapse and the release of hydrolytic enzymes such as proteases and nucleases that rapidly dismantle cellular components (Pajerowska‐Mukhtar and Dong 2009). In *P. syringae* ‐infected leaves, synthetic caspase‐1 and caspase‐3 inhibitors markedly suppress HR lesions, demonstrating that VPE activity confines biotrophic pathogens (del Pozo and Lam 1998). Similarly, TMV‐induced HR depends on VPE‐mediated vacuolar collapse, as confirmed by virus‐induced gene silencing (VIGS) (Hatsugai et al. 2004). Conversely, the necrotroph *Fusarium moniliforme* exploits this pathway by secreting fumonisin B1 to trigger VPE‐dependent death in *Arabidopsis* (Kuroyanagi et al. 2005). When unresolved ER stress overrides adaptive UPR, γVPE becomes the terminal executor of ER‐PCD. The mutualistic fungus *Piriformospora indica* colonises *Arabidopsis* roots by inducing ER stress while repressing adaptive UPR, thereby forcing γVPE activation and cell death (Qiang et al. 2012). This phenocopies Tm‐induced cell death, as both stimuli elevate γVPE activity and caspase‐1‐like activity (Qiang et al. 2012). Thus, VPEs function as a conserved execution node where HR‐PCD and ER‐PCD converge on vacuole rupture. The integration of HR and ER‐PCD is further orchestrated by a set of regulatory proteins that sense both pathogen presence and ER homeostasis. Plants lack orthologs of Bcl‐2 or BAX/BAK but retain BI‐1 (Bax Inhibitor‐1) and BAG (Bcl‐2 associated athanogene) proteins that modulate ER stress output (Oh et al. 2003; Xu and Reed 1998). BI‐1 is a conserved gatekeeper that restrains ER‐PCD and serves as a tunable switch between survival and death, responding to stress duration and intensity (Watanabe and Lam 2008b; Ruberti et al. 2018). In pathosystems, this PCD‐suppressive function of BI‐1 can be exploited. In barley, HR cell death triggered by deletion of the *Ustilago maydis* effector *Pep1* can be rescued by *BI‐1* overexpression, confirming BI‐1 as a susceptibility factor that preserves host cell viability for biotrophs (Hof et al. 2014). The endophyte *Piriformospora indica* down‐regulates *BI‐1* to sustain colonisation as evidenced by the fact that transgenic barley overexpressing *BI‐1* blocks fungal proliferation in roots (Deshmukh et al. 2006). During *Phytophthora* challenge, BAG7 exerts a location‐dependent switch between susceptibility and resistance. In the ER, BAG7 binds BiP and is retained there; this interaction represses VPE‐driven PCD and weakens basal immunity (Zhou et al. 2021). Once BAG7 escapes BiP and enters the nucleus, it initiates ER‐PCD and upregulates defence genes, markedly enhancing resistance (Zhou et al. 2021). NAC089 is a master switch that initiates ER‐PCD during pathogen challenge and confers broad resistance to *P. capsici*, *P. syringae* , TMV and CMV (Shen et al. 2017; Li et al. 2018; Ai et al. 2021). Upon perception of *Phytophthora*, nuclear NAC089 activates caspase‐3‐like proteases and PCD‐executing genes to eliminate infected cells (Ai et al. 2021), while during viral infection, it selectively upregulates PCD genes and dampens UPR activation to generate an antiviral environment that curtails pathogen spread (Li et al. 2018). The pro‐death activity of NAC089 is modulated by UPR components IRE1 and BAP2, which fine‐tune its expression to ensure that PCD is deployed only when ER stress exceeds adaptive capacity (Pastor‐Cantizano et al. 2024). Beyond this role in calibrating ER‐PCD, BAP2 also functions more broadly as a suppressor of pathogen‐triggered HR and oxidative death, although single mutants do not gain resistance to virulent strains (Yang et al. 2007). While direct evidence linking BAP2 to ER‐PCD during infection remains scarce, these data imply a shared regulatory axis between ER stress and pathogen defence.

In summary, HR‐PCD and ER‐PCD are not isolated pathways but rather represent two arms of an integrated cell death response. Vacuolar Processing Enzymes (VPEs) serve as the central execution node, mediating vacuolar collapse in both contexts. Regulatory proteins like BI‐1, BAG7 and NAC089 sit at the interface, integrating pathogen‐derived and ER stress‐derived signals to calibrate the final cell fate decision.

#### 4.3.2 Quality Control Factors as Gatekeepers of Pathogen‐Triggered PCD

Beyond the dedicated PCD regulators discussed above, the general machinery that maintains ER homeostasis—particularly molecular chaperones and folding factors—functions as a critical gatekeeper that determines whether pathogen‐triggered cell death programs are permitted to proceed.

The ER chaperone BiP exemplifies how the same factor can exert opposing effects on PCD depending on cellular context. In soybean and tobacco, *BiP* overexpression accelerates non‐host HR against *P. syringae* , triggering NRP‐ and VPE‐dependent death downstream of SA‐induced ER stress (Carvalho et al. 2014). Consistently, BiP and CRT3 stabilise Cf‐dependent HR in *N. benthamiana* and silencing *CRT2/3* weakens HR, allowing bacterial proliferation (Caplan et al. 2009). CRT3, together with ERdj3b and SDF2, stabilises SOBIR1, a positive regulator of cell death that promotes the constitutive defences and PCD observed in *Arabidopsis bir1* mutants (Sun et al. 2014). In these contexts, enhanced chaperone capacity appears to support the production and stability of PCD‐executing machinery, which requires rapid synthesis and folding of immune components. By contrast, elevated *BiP* suppresses PVX TGBp3‐triggered ER‐PCD, preserving tissue integrity and facilitating viral movement (Ye et al. 2011). During *Phytophthora* challenge, ectopic *GmBiP1/4* or *NbBiP5* expression blunts BAX‐induced ER‐PCD, limiting pathogen ingress (Jing et al. 2016). Loss of *BiP2* in *Arabidopsis* uncouples SA‐driven PR overproduction from folding capacity, leading to lethal ER stress; the *bip2 npr1* double mutant confirms that NPR1‐dependent PR accumulation is the driver of this death (Wang et al. 2005). Thus, BiP‐mediated proteostasis can either amplify or buffer PCD outputs, depending on whether the death program relies on newly synthesised immune components (as in HR) or is driven by proteotoxic overload (as in ER‐PCD).

By retrieving HDEL/KDEL‐bearing luminal proteins, ERD2a and ERD2b sustain the chaperone triad BiP, CRT3 and UGGT, which are required to buffer ER stress (Xu et al. 2012). Silencing assays expose non‐redundant and even antagonistic roles: *ERD2b* knock‐down postpones the BiP‐dependent HR triggered by *Xanthomonas oryzae* and the CRT3/UGGT‐dependent HR evoked by *Pseudomonas*, whereas *ERD2a* single or combined knock‐down accelerates PCD after either *Xoo* or *Pst* DC3000 challenge (Xu et al. 2012). These opposing phenotypes suggest a dose‐dependent threshold model: a certain level of chaperone supply and retrograde trafficking is required to support HR execution, but excessive ER retention of chaperones may hyper‐suppress stress signalling and delay necessary cell death, while insufficient retrieval leads to chaperone depletion and premature proteotoxicity‐driven death.

### 4.4 Induction of ERAD

The emerging evidence reveals that UPR‐ERAD coordination is critical for plants to mount robust immune responses without succumbing to ER proteotoxicity (Figure 6E).

A well‐characterised function of ERAD in immunity is the quality control of PRRs. Multiple ERAD components have been implicated in the degradation of the misfolded EFR receptor, including the E3 ligase components HRD1A/HRD1B, the lectin‐like adaptors EBS6/AtOS9, the plant‐specific EBS7, and the PAWH1/PAWH2 complex (Lin et al. 2019; Liu et al. 2015; Su et al. 2012). These components cooperatively recognise and retrotranslocate misfolded EFR from the ER lumen to the cytosol for proteasomal degradation, thereby maintaining the fidelity of PRR‐mediated immune surveillance. Notably, the expression of several ERAD components, such as *AtOS9*, *EBS7* and *PAWH1/PAWH2*, is directly upregulated by ER stress inducers like Tm, positioning them as bona fide UPR target genes (Lin et al. 2019; Liu et al. 2015; Su et al. 2012). Moreover, defects in ERAD function lead to accumulation of misfolded proteins in the ER lumen, which in turn activates UPR signalling as a compensatory response. The *Arabidopsis os9*, *ebs7* and *pawh1 pawh2* mutants, which lack key ERAD components, exhibit constitutive UPR activation and hypersensitivity to ER stress agents such as DTT and Tm (Lin et al. 2019; Liu et al. 2015; Su et al. 2012). These observations reveal an elegant homeostatic circuit that UPR transcriptionally induces ERAD components to enhance degradation capacity, while ERAD dysfunction feeds back to activate UPR, ensuring robust maintenance of ER homeostasis under the fluctuating demands imposed by pathogen infection.

Beyond its quality control and homeostatic functions, the ERAD system can directly contribute to immune activation by targeting negative regulators for degradation. In rice, the ERAD component OsUBC45 functions as an E2 ubiquitin‐conjugating enzyme and confers broad‐spectrum disease resistance against both the fungal pathogen *Magnaporthe oryzae* and the bacterial pathogen *Xanthomonas oryzae* (Wang et al. 2023). Upon pathogen infection or treatment with the PAMP chitin, *OsUBC45* expression is strongly induced—a response that coincides with activation of the UPR, suggesting that ER stress is engaged during immune activation (Wang et al. 2023). OsUBC45 then promotes degradation of the aquaporin OsPIP2; 1—a validated negative regulator of PTI—thereby relieving immune suppression and enhancing basal resistance (Wang et al. 2023). Whether UPR signalling directly regulates *OsUBC45* expression, or whether additional UPR‐dependent mechanisms contribute to ERAD‐mediated immunity, remains an important question for future investigation.

The UPR‐ERAD interface is not a simple linear pathway but a dynamically balanced network. Studies on the *Arabidopsis* UBC32/33/34 E2 enzyme family reveal sophisticated feedback regulation (Wang et al. 2025). UBC32, UBC33, and UBC34 are ER‐localised E2 enzymes in the ERAD system with partially overlapping yet antagonistic functions. UBC32 appears to act as a brake on UPR‐ERAD output, while UBC33/34 function as accelerators by promoting degradation of regulatory factors including UBC32 itself (Wang et al. 2025). Consequently, *ubc33/34* mutants accumulate UBC32, leading to excessive UPR suppression and impaired ER stress adaptation, which renders plants hypersusceptible to *Pseudomonas syringae* infection (Wang et al. 2025). This antagonistic relationship ensures that UPR‐ERAD activity is precisely titrated—sufficient to clear misfolded immune proteins but restrained to prevent hyperactivation that could disrupt ER homeostasis or deplete cellular resources.

Compared with mammalian systems, where ERAD has been extensively linked to immune receptor turnover and pathogen evasion strategies, direct evidence for the regulatory circuits connecting UPR activation to specific ERAD components during plant‐pathogen interactions is still accumulating. Elucidating whether and how UPR modulates ERAD under immune challenge, and identifying additional ERAD substrates that function as immune regulators, represents important directions for future research.

### 4.5 Induction of Selective Autophagy

Beyond the canonical role in eliminating misfolded protein aggregates and damaged ER fragments (Liu et al. 2012; Munch et al. 2014; Zeng et al. 2019; Bao and Bassham 2020), ER‐phagy encompasses diverse functions in plant immunity, ranging from passive damage clearance to active immunomodulation (Figure 6D) (Hofius et al. 2017; Jeon et al. 2025; Ahmed et al. 2026). Importantly, while ER‐phagy specifically refers to autophagy of the ER itself, the UPR also regulates broader selective autophagy pathways that collectively contribute to plant defence (Li et al. 2020).

A paradigm‐shifting discovery established ER‐phagy as a proactive immune mechanism rather than merely a damage‐control system. Upon *M. oryzae* infection or ER stress induction, the ER‐phagy receptor OsHLP1 simultaneously binds its cargo OsNTL6 and the autophagy machinery component OsATG8b, facilitating the formation of ER‐derived autophagosomes that deliver OsNTL6 to the vacuole for degradation, thereby relieving transcriptional repression of defence genes and activating rice immunity against the fungal pathogen (Liang et al. 2023). Notably, this pathway is intimately linked to UPR activation: *M. oryzae* infection damages the rice ER network—a morphological hallmark of ER stress—and chitin‐induced UPR gene expression is largely dependent on *OsHLP1* (Meng et al. 2022). Thus, OsHLP1‐mediated ER disruption triggers UPR activation, which serves as a molecular bridge converting physical ER damage into biochemical signals that ultimately de‐repress immunity.

In viral infections, ER‐phagy exhibits a more complex duality, functioning as both a host restriction mechanism and, in some contexts, a process co‐opted by viruses. The UPR acts as a central orchestrator of these opposing outcomes through its downstream ER‐phagy effectors. During Beet black scorch virus (BBSV) infection, the viral protein p23 induces ER stress, activating UPR to create a proviral environment that facilitates viral replication; indeed, pharmacological induction of UPR (e.g., by DTT or Tm) significantly enhances BBSV accumulation, whereas silencing the UPR sensor IRE1b suppresses it (Wang et al. 2026). At the same time, ER‐phagy mediated by the receptor Sec62 is triggered in an IRE1‐dependent manner, selectively degrading viral replication organelles (VROs), which are aberrant ER membrane structures hijacked by the virus for replication (Wang et al. 2026). This ‘self‐restraint’ mechanism not only restricts BBSV but also confers broad‐spectrum resistance against other ER‐replicating positive‐strand RNA viruses, including TMV and TuMV, revealing the UPR‐ER‐phagy axis as a conserved antiviral checkpoint (Wang et al. 2026). A comparable duality of ER‐phagy characterises the bZIP28/bZIP17 pathway. *Geminiviruses* deploy the ER‐anchored βV1 viral protein to trigger ER aggregation, thereby activating bZIP28/bZIP17 to synthesise viral proteins and enhance viral infectivity (Hu et al. 2024). Plants retaliate with ATG18a‐mediated ER‐phagy—paradoxically initiated by bZIP28/bZIP17 signalling itself—to eliminate βV1 and restrict the virus (Hu et al. 2024).

Beyond ER‐phagy, the UPR also orchestrates broader selective autophagy pathways that directly target viral proteins. During TuMV infection, the viral 6 K2 protein activates the IRE1/bZIP60 pathway, which upregulates the autophagy receptor NBR1. In a striking twist, instead of targeting the virus for degradation, NBR1 is co‐opted to recruit ATG8f to sequester viral replication vesicles from autolysosomal destruction, inadvertently furnishing membrane niches that foster viral replication near the tonoplast (Figure 6D) (Li et al. 2020). Conversely, NBR1 also exerts antiviral functions: it binds to the P4 protein of Cauliflower mosaic virus (CaMV), targeting it for autophagic degradation and thereby restricting viral infection (Hafrén et al. 2017). Thus, NBR1‐mediated selective autophagy can either limit or promote viral infection depending on the viral context and the specific cargo targeted. Rice stripe virus (RSV) selectively activates the bZIP17/28 UPR branch via its membrane proteins NSvc2 and NSvc4, which stabilise and promote the proteolytic processing and nuclear translocation of bZIP28/bZIP17, thereby facilitating viral replication (Li et al. 2022). The bZIP17 activation simultaneously upregulates host autophagy, which targets the viral protein NSvc4 for degradation (Li et al. 2021). To counterbalance this restriction, RSV employs the host protein NbMIP1s to protect NSvc4 from complete autophagic clearance through direct protein interaction, thereby forming an ‘activate‐limit‐protect’ regulatory loop that fine‐tunes viral proliferation (Figure 6D) (Li et al. 2021).

BI‐1 adds another regulatory layer to plant immunity by functioning as an ER‐localised autophagic rheostat that determines cell fate under ER stress. BI‐1 physically interacts with ATG6 at the ER membrane, thereby recruiting core autophagy machinery to initiate ER‐originating autophagosome formation (Xu et al. 2017). During N gene‐mediated resistance to TMV, BI‐1 promotes pro‐survival autophagy; silencing *BI‐1* significantly compromises this immune‐induced autophagy and accelerates HR‐PCD (Xu et al. 2017). Overexpressing *BI‐1*, however, increases basal autophagy yet sensitises tissues to stress‐triggered, autophagy‐dependent death (Xu et al. 2017). Thus, BI‐1 exhibits a dose‐dependent functional duality: at endogenous levels, it sustains cytoprotective ER‐autophagy during immune activation, whereas its overaccumulation switches the output to lethal, self‐amplifying ER‐autophagy, positioning it as a decisive molecular toggle in plant immune cell fate decisions.

Collectively, this UPR‐autophagy axis thus exemplifies how plants have evolved to harness organelle turnover not merely for damage control, but as a strategically deployed weapon in immune defence—one that pathogens in turn have learned to exploit.

## 5 Pathogen Hijacking of the Host ER Stress Response

The ER stress response has become a central battlefield in plant‐pathogen co‐evolution, where each side seeks to control its regulatory circuitry (Figure 6) (Afrin et al. 2020; Adhikari et al. 2025).

Phytopathogens have evolved intricate molecular arsenals to rewire the host ER stress circuitry, converting a potentially lethal defence program into a gateway for colonisation. A recurring tactic is the translocation of effector proteins across the host plasma membrane to directly modulate master regulators of the UPR (Breeze et al. 2020; Kørner et al. 2015). Notably, UPR marker genes typically upregulated by PAMPs remain unchanged—or are even downregulated—during infection by *P. capsici* (Tateda et al. 2008). Similarly, in bacterial infection contexts, the UPR is activated in response to the non‐host pathogen *Pseudomonas cichorii* , but not upon infection with the host‐adapted pathogen *P. syringae* (Tateda et al. 2008). These findings suggest that successful pathogens may have undergone convergent evolution to acquire the ability to suppress ER stress‐mediated plant immunity by targeting key components of the UPR and perturbing ER homeostasis through effector activity. During the early, biotrophic phase of hemibiotrophic infections, oomycetes such as *Phytophthora* spp. transiently provoke ER stress, an event that can culminate in PCD and thereby arrest pathogen ingress. To circumvent this checkpoint and establish a stable feeding site, *P. sojae* secretes the RxLR effector PsAvh262, which orchestrates a multi‐pronged suppression of both pro‐death and immune outputs (Jing et al. 2016; Zhou et al. 2021). PsAvh262 binds and shields the ER chaperone BiP from 26S‐proteasome‐mediated degradation, leading to an aberrant accumulation of BiP in the ER lumen (Jing et al. 2016). Elevated BiP levels sequester two key UPR transducers—BAG7 and bZIP28—trapping them within the ER and preventing their nuclear mobilisation (Figure 6A) (Zhou et al. 2021). In the nucleus, BAG7 normally amplifies ER‐stress‐induced immunity (ERSI) and PCD; its ER retention converts BAG7 into a susceptibility factor that dampens ROS bursts, downregulates defence genes (e.g., *VPE*, *SGT1*, *PR1*), and blocks infection‐induced ER‐PCD (Zhou et al. 2021). Similarly, retention of bZIP28 cripples the canonical UPR transcriptional cascade that would otherwise bolster resistance (Zhou et al. 2021). A parallel strategy is executed by *P. capsici*. Its RxLR effector PcAvr3a12 physically associates with FKBP15‐2, an ER‐resident peptidyl‐prolyl cis‐trans isomerase (PPIase) that catalyses the rate‐limiting proline isomerisation required for proper protein folding and ER stress sensing (Fan et al. 2018). By inhibiting FKBP15‐2 PPIase activity, PcAvr3a12 attenuates the transcriptional upregulation of the ER‐stress sensors *bZIP28* and *bZIP60*, crippling the UPR and rendering the host hypersusceptible to *P. capsici* infection (Figure 6A) (Fan et al. 2018). Collectively, these examples illustrate how effector‐mediated hijacking of ER stress nodes allows pathogens to convert a plant fail‐safe mechanism into a vulnerability.

Plant viruses have evolved sophisticated tactics to commandeer the host UPR by reprogramming ER stress sensors. These manoeuvres enhance viral protein folding, suppress defence‐associated PCD, and redirect cellular resources toward pathogen replication. A common strategy across diverse viral taxa is the use of viral proteins to selectively activate specific UPR branches. For example, the betasatellite‐encoded βC1 protein of the geminivirus complex TYLCCNV/TYLCCNB selectively manipulates the IRE1–bZIP60 axis to amplify viral propagation (Zhang et al. 2023). Upon infection, βC1 rapidly elevates *bZIP60* transcript levels and enforces a precise spatiotemporal itinerary of the transcription factor. During early infection, nuclear accumulation of bZIP60 drives the expression of ER‐resident chaperones such as *BiP* and *CRT*, thereby ensuring correct folding and high‐level accumulation of viral proteins (Zhang et al. 2023). To avert a late, cytotoxic surge of ER stress that could trigger NAC089‐mediated PCD, βC1 later escorts bZIP60 from the nucleus back to the cytoplasm, thereby preventing the activation of NAC089 in the nucleus (Figure 6B,C) (Zhang et al. 2023). This dynamic nucleocytoplasmic shuttling converts the host UPR into a stage‐by‐stage support platform for viral survival and amplification. Parallel strategies are observed across diverse viral taxa. The pro‐viral centrality of bZIP60 is corroborated by markedly reduced titres and attenuated disease severity in *bZIP60*‐silenced hosts infected with PVX, Rice black‐streaked dwarf virus (RBSDV), or Garlic virus X (Ye et al. 2011; Sun, Yang, Xie, et al. 2013; Lu et al. 2016). Similarly, Beet necrotic yellow vein virus (BNYVV) induces the susceptibility factor VOC1, which translocates to the nucleus and physically associates with bZIP28/bZIP17 (Guo et al. 2024). These interactions potentiate the DNA‐binding activity of bZIP28 and bZIP17, further amplifying UPR signalling. In turn, bZIP28/bZIP17 reciprocally stimulates *VOC1* transcription, establishing a self‐reinforcing pro‐viral feedback loop that markedly enhances BNYVV accumulation (Figure 6B) (Guo et al. 2024). This principle extends to other viruses as well, where viral proteins such as BBSV p23, geminivirus βV1, TuMV 6K2 and RSV NSvc2/4 actively activate specific UPR branches to create a pro‐viral replication environment (Hu et al. 2024; Li et al. 2020, 2022; Wang et al. 2026).

Finally, certain viruses bypass canonical ER stress sensors entirely. Soybean mosaic virus (SMV) employs the P3 virulence protein to directly hijack the host translational elongation machinery by interacting with the eEF1A–eEF1B complex (Luan et al. 2016). Both *GmEF1A* and *GmEF1B* are indispensable for P3‐triggered UPR induction. By tethering P3 to *eEF1A*, the virus positions the translation elongation factor upstream of *BiP* and other canonical UPR sensors, thereby orchestrating an ER environment that favours SMV replication over host defence (Figure 6B) (Luan et al. 2016). Thus, P3‐mediated subversion of eEF1A‐dependent elongation represents a non‐canonical route to ER stress that paradoxically promotes viral virulence.

Across kingdoms, pathogens have convergently weaponised the ER stress circuitry as a shared susceptibility hub. Pathogens may deploy a broader array of factors beyond known effectors or virulence proteins to disrupt sensors, rewire transcription, or induce non‐canonical UPR, thereby turning a primordial safeguard into a pro‐colonisation niche that dismantles ER immunity and accelerates proliferation.

## 6 A Conceptual Framework for Context‐Dependent UPR Outcomes

**FIGURE 7:**
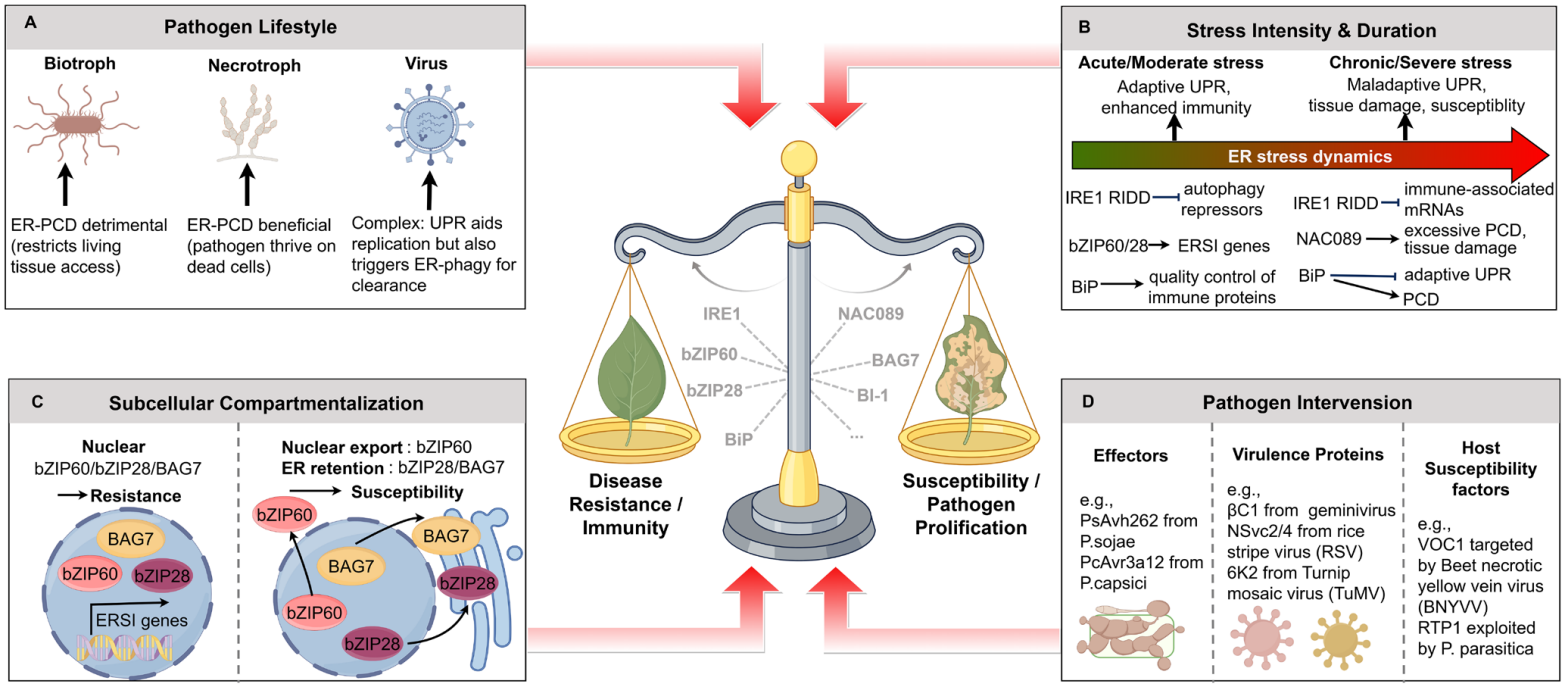
A conceptual framework for context‐dependent UPR outcomes in plant‐pathogen interactions. UPR activation can tip the balance toward either resistance (left) or susceptibility (right) depending on the context of plant‐pathogen interaction. Four key variables jointly shape the outcome; the examples shown within each module are representative and not exhaustive. (A) Pathogen lifestyle. ER‐PCD restricts biotrophic pathogens by eliminating living host tissue but benefits necrotrophs that thrive on dead cells. For viruses, UPR‐enhanced folding can promote replication, whereas ER‐phagy can restrict infection. (B) Stress intensity and duration. Acute, moderate ER stress promotes adaptive UPR programs that enhance protein quality control, defence genes activation and controlled PCD. Chronic or severe stress shifts the balance toward maladaptive outcomes, including excessive RIDD‐mediated decay of immune‐associated mRNAs, runaway NAC089‐driven tissue damage, and BiP‐mediated sequestration of UPR sensors. (C) Subcellular compartmentalisation. Nuclear localisation of bZIP60, bZIP28, and BAG7 activates ERSI and promotes resistance. Cytoplasmic sequestration or ER retention of these regulators compromises defence activation. (D) Pathogen intervention. Effectors (e.g., PsAvh262, PcAvr3a12), viral proteins (e.g., βC1, NSvc2/4, 6K2), and co‐opted host susceptibility factors (e.g., VOC1, RTP1) recalibrate UPR boundary conditions, converting host ER stress circuitry from a defensive asset into a niche permissive for pathogen colonisation.

Activation of the same ER stress module can lead to diametrically opposed outcomes—resistance or susceptibility—depending on infection context. Rather than reflecting inconsistent biology, this suggests that UPR outputs are not predetermined but are likely calibrated by a set of core boundary conditions. By integrating evidence from multiple UPR components, including the core signalling pathways IRE1‐bZIP60, bZIP28/bZIP17 and IRE1‐RIDD, key regulatory nodes such as BiP, NAC089, BAG7 and BI‐1, and downstream processes such as ER‐associated PCD, ERAD, and ER‐phagy, we propose a conceptual framework in which the final outcome is shaped by four key variables (Figure 7).

First, pathogen lifestyle serves as the primary determinant that sets the ‘rules of engagement’ (Figure 7A). The functional consequence of any UPR‐driven process is intrinsically linked to whether the invading pathogen is a biotroph, necrotroph or virus. UPR outputs that culminate in ER‐PCD are generally beneficial against biotrophic and hemibiotrophic pathogens, as PCD restricts their access to living tissues and curtails infection spread (Carvalho et al. 2014; Yang, Wang, et al. 2014; Moon et al. 2016; del Pozo and Lam 1998). Conversely, the very same PCD execution can be detrimental during necrotrophic infection, where pathogens thrive on dead cells and actively exploit host cell death machinery for nutrient acquisition (Qiang et al. 2012; Kuroyanagi et al. 2005). For viruses, the equation is further complicated by their absolute dependence on host cellular machinery: UPR‐enhanced folding capacity can stabilise viral proteins and promote replication (Ye et al. 2011; Zhang et al. 2023), while UPR‐triggered ER‐phagy can selectively degrade viral components and restrict infection (Li et al. 2020; Hu et al. 2024). Thus, pathogen lifestyle dictates whether a given UPR output is interpreted by the plant as a defensive asset or an offensive liability.

Second, the intensity and duration of ER stress function as a rheostat that toggles between adaptive and maladaptive outcomes (Figure 7B). Acute, moderate ER stress—often triggered by successful PAMP perception during early infection—promotes adaptive UPR programs that bolster immunity. Under these conditions, IRE1‐RIDD may selectively degrade autophagy repressors to maintain cellular homeostasis (Bao et al. 2018), bZIP60 and bZIP28 activate ERSI gene programs (Moreno et al. 2012; Arraño‐Salinas et al. 2018), and BiP supports the maturation of immune receptors and secretion of defence proteins (Li et al. 2009; Nekrasov et al. 2009). In contrast, chronic or severe ER stress may shift the balance toward maladaptive outcomes: sustained IRE1‐RIDD could degrade immune‐associated mRNAs, compromising defence signalling (Mishiba et al. 2013); BiP hyperaccumulation inactivates UPR sensors through direct binding, dampens adaptive responses, and potentiates NRP‐mediated PCD (Jing et al. 2016; Liu and Howell 2010); and NAC089‐mediated PCD transitions from a controlled defence to uncontrolled tissue damage that can facilitate pathogen colonisation (Yang, Wang, et al. 2014). These findings raise the possibility that the same molecular machinery may have different functional consequences depending on stress dynamics.

Third, the specific UPR branch engaged and its subcellular compartmentalisation critically influence outcome directionality by determining which downstream effectors are activated (Figure 7C). The IRE1‐bZIP60 module exemplifies this principle: nuclear‐localised bZIP60 activates ERSI and promotes resistance to bacteria and fungi (Moreno et al. 2012; Xu et al. 2019), whereas ER‐retained or cytoplasmically sequestered bZIP60, whether through effector or viral protein manipulation such as *geminivirus* βC1‐mediated nuclear export or through susceptibility factors such as RTP1 stabilisation, fails to activate defence programs and instead becomes a liability that pathogens exploit (Qiang et al. 2021; Zhang et al. 2023). Similarly, BAG7 requires nuclear translocation to engage WRKY29‐dependent immunity; its effector‐driven retention in the ER converts it from a transcriptional co‐activator into a susceptibility factor that actively suppresses ERSI (Zhou et al. 2021). The functional dichotomy of autophagy further illustrates this point: ATG18a‐mediated ER‐phagy eliminates viral proteins and restricts infection (Hu et al. 2024), whereas NBR1‐ATG8f‐mediated autophagy can shield viral replication complexes from degradation, furnishing a protected niche for viral propagation (Li et al. 2020). Thus, the same core machinery can be channelled toward defence or co‐opted for pathogenesis depending on which downstream effectors are engaged and where they are localised within the cell.

Fourth, pathogen effectors and virulent proteins can actively manipulate these boundary conditions to tip the balance decisively toward susceptibility (Figure 7D). Across kingdoms, pathogens have evolved convergent strategies to hijack UPR components, effectively recalibrating the host's stress response machinery for their own benefit, as detailed in Section 5. Oomycete effectors (e.g., PsAvh262 from *P. sojae* [Jing et al. 2016; Zhou et al. 2021], PcAvr3a12 from *P. capsici* [Fan et al. 2018]), viral proteins (e.g., geminivirus βC1 [Zhang et al. 2023], RSV NSvc2/4 [Li et al. 2022]), and host susceptibility factors exploited by pathogens (e.g., RTP1 [Qiang et al. 2021], VOC1 [Guo et al. 2024]) have all been shown to target specific UPR nodes. Rather than simply suppressing UPR, these pathogen‐derived or pathogen‐manipulated factors strategically reprogram the boundary conditions themselves, converting the ER from a defensive stronghold into a permissive niche for pathogen colonisation.

This framework offers a way to reconcile seemingly contradictory reports in the literature by positioning them not as anomalies but as predictable outcomes under specific boundary conditions. The same UPR component can appear as a resistance factor in one study and a susceptibility factor in another depending on where the experimental system fell within this matrix. This conceptual synthesis highlights why high‐resolution spatiotemporal maps of UPR activity—capturing stress dynamics, subcellular localisation, and pathogen manipulation across different infection stages and pathogen types—are urgently needed: only by understanding how these variables interact in real‐time can we predict UPR outcomes with confidence.

## 7 Conclusion and Perspectives

### 7.1 Concluding Remarks and Future Perspectives on Plant‐Pathogen Interactions

This review consolidates emerging evidence that the UPR is a pivotal interface between host defence and pathogen offence. We discuss the two dominant UPR branches, IRE1‐bZIP60 and bZIP28/bZIP17, and show how they integrate with PTI, ETI, SAR, and ISR. UPR components emerge as active immune drivers, rather than passive stress gauges: they support the folding and secretion of defence proteins, tune transcriptional and translational programs, calibrate PCD, and trigger ERAD and autophagy. A recurrent motif is the struggle for ER control—plants weaponise the UPR for pathogen detection and containment, while fungi, oomycetes, and viruses deploy effectors to hijack, dampen, or repurpose the same circuitry. This ongoing co‐evolution positions the ER as the decisive signalling hub that dictates infection outcomes. Synthesising these observations, we propose a conceptual framework in which the outcome of this struggle—resistance versus susceptibility—is shaped by the interplay of pathogen lifestyle, ER stress dynamics, subcellular compartmentalisation and pathogen intervention.

While this framework provides a valuable lens for reconciling seemingly contradictory findings, it also underscores profound gaps in our understanding. Whether additional variables remain to be identified, the precise molecular mechanisms by which these variables interact are largely elusive—particularly how plants discriminate pathogen‐triggered ER perturbations from abiotic insults, and how pathogens dynamically reprogram UPR outputs across infection stages. Furthermore, we still lack a mechanistic understanding of how basal UPR activity, which operates even in the absence of stress to support development, dynamically calibrates the long‐term trade‐off between growth and pathogen defence. Elucidating how plants fine‐tune UPR activation to balance these competing demands under fluctuating environmental pressures represents a key conceptual frontier. Moreover, the complete inventory of pathogen effectors that target UPR nodes and their modes of action remains fragmentary. Closing these knowledge gaps will open practical avenues: small molecules or RNAi constructs that neutralise UPR‐directed virulence factors, and genetic engineering that reinforces ER homeostasis to simultaneously enhance disease resistance and abiotic stress tolerance, thereby safeguarding crop yield under future climate scenarios.

### 7.2 UPR in Crop Biotechnology: Mitigating Confounding Effects and Harnessing Opportunities

The principles of UPR signalling elucidated through plant‐pathogen interactions present both challenges and opportunities for plant biotechnology, in contexts ranging from stable transgenic crops to transient expression systems used in plant molecular farming. A critical yet overlooked consideration is that high‐level or constitutive transgene expression can itself act as a persistent ER stressor (Hamel et al. 2024; Li, Yang, et al. 2025), potentially confounding the interpretation of engineered traits or limiting product yield. For instance, phenotypes attributed to the overexpression of immune components may not solely result from enhanced immunity but may also arise, in part, from generalised UPR activation, which can produce nonspecific resistance accompanied by growth penalties, often referred to as a ‘yield penalty’. Critically, the relationship between UPR activation and bioproduction outcome is dynamic: transient and moderate UPR correlates with high product yields, whereas severe or chronic UPR activation leads to poor output and cellular damage (Hamel et al. 2024). This underscores that precise management of UPR activity, with controlled and timely activation rather than blanket suppression, is key to unlocking its potential.

Integrating UPR management into plant biotechnological design is therefore imperative and requires a dual strategy aimed at minimising its confounding effects while harnessing its beneficial potential. This can be achieved through two complementary approaches: mitigating unintended UPR activation and intentionally harnessing UPR components. To address the former, the use of tissue‐specific or stress‐inducible promoters can minimise constitutive ER loading, while co‐expression of chaperones can enhance folding capacity. For instance, co‐expressing *Arabidopsis*‐derived chaperones such as *BiP*, *CRT* or *CNX* has been shown to increase the overall yield of the Immunoglobulin A in *Nicotiana benthamiana*, by facilitating the assembly or stabilisation of its heavy and light chains (Goritzer et al. 2020). To address the latter, deliberate manipulation of UPR nodes offers a direct route to enhance both crop traits and recombinant protein yields. For example, co‐expression of *bZIP28* markedly enhances the accumulation of complex therapeutic proteins like human erythropoietin (hEPO) in plant systems (Wagner et al. 2024). Similarly, strategic modulation of the QT12–IRE1 or RISBZ1 modules in rice can rebalance storage metabolism, stabilising grain quality under heat stress and improving grain filling (Li, Yang, et al. 2025; Sun et al. 2025).

To translate these strategic principles into practice and ensure that observed phenotypes are attributable to specific mechanisms, future experimental designs should adopt a set of diagnostic and stratified approaches. First, the routine monitoring of core UPR markers—such as *bZIP60* mRNA splicing, bZIP28 protein processing, and the transcript levels of chaperones like *BiP*, *PDI* and *CRT*—should be established as a standard. This serves as a quantitative measure of the ‘UPR dosage’ elicited by transgene expression. Second, employing inducible rather than constitutive promoters provides temporal control over ER load. Generating and analysing a series of transgenic lines with graded expression levels of the transgene allows researchers to correlate the magnitude of UPR activation with the intended phenotypic outcome and any growth penalties. Alternatively, the application of chemical chaperones such as TUDCA to alleviate ER stress can be used to test whether a phenotype is reversible and therefore likely linked to proteostasis imbalance. This disciplined framework for UPR‐aware biotechnological design will ensure that observed improvements in crop performance or recombinant protein yield can be confidently attributed to intended mechanisms rather than unresolved ER stress, thereby unlocking the full potential of plant biotechnology to deliver high‐performing, resilient crops and efficient plant biofactories.

## Author Contributions

All authors contributed to the preparation of the manuscript. Z.M., F.B. and X.Z. conceptualised the review outline. Z.M., S.Z., Y.L., C.A. and Y.W. wrote the original draft; S.Z., Y.L., J.Q. and C.L. prepared the figures; F.B. commented and revised the manuscript; X.Z. reviewed and edited the manuscript. The authors declare that there are no conflicts of interest.

## Conflicts of Interest

The authors declare no conflicts of interest.

## Data Availability

The data that support the findings of this study are available on request from the corresponding author. The data are not publicly available due to privacy or ethical restrictions.
